# Evidence of previous but not current transmission of chikungunya virus in southern and central Vietnam: Results from a systematic review and a seroprevalence study in four locations

**DOI:** 10.1371/journal.pntd.0006246

**Published:** 2018-02-09

**Authors:** Tran Minh Quan, Huynh Thi Phuong, Nguyen Ha Thao Vy, Nguyen Thi Le Thanh, Nguyen Thi Nam Lien, Tran Thi Kim Hong, Pham Ngoc Dung, Nguyen Van Vinh Chau, Maciej F. Boni, Hannah E. Clapham

**Affiliations:** 1 Mathematical Modelling Department, Oxford University Clinical Research Unit, Wellcome Trust Major Overseas Programme, Ho Chi Minh City, Vietnam; 2 Microbiology Department, Hue Central Hospital, Hue, Thua Thien Hue province, Vietnam; 3 Laboratory Department, Dak Lak General Hospital, Buon Ma Thuot, Vietnam; 4 Laboratory Department, An Giang General Hospital, An Giang province, Vietnam; 5 Hospital for Tropical Diseases, Ho Chi Minh City, Vietnam; 6 Centre for Tropical Medicine and Global Health, Nuffield Department of Medicine, University of Oxford, Oxford, United Kingdom; 7 Center for Infectious Disease Dynamics, Department of Biology, Pennsylvania State University, University Park, Pennsylvania, United States of America; Institute for Disease Modeling, UNITED STATES

## Abstract

**Background:**

Arbovirus infections are a serious concern in tropical countries due to their high levels of transmission and morbidity. With the outbreaks of chikungunya (CHIKV) in surrounding regions in recent years and the fact that the environment in Vietnam is suitable for the vectors of CHIKV, the possibility of transmission of CHIKV in Vietnam is of great interest. However, information about CHIKV activity in Vietnam remains limited.

**Methodology:**

In order to address this question, we performed a systematic review of CHIKV in Vietnam and a CHIKV seroprevalence survey. The seroprevalence survey tested for CHIKV IgG in population serum samples from individuals of all ages in 2015 from four locations in Vietnam.

**Principal findings:**

The four locations were An Giang province (n = 137), Ho Chi Minh City (n = 136), Dak Lak province (n = 137), and Hue City (n = 136). The findings give us evidence of some CHIKV activity: 73/546 of overall samples were seropositive (13.4%). The age-adjusted seroprevalences were 12.30% (6.58–18.02), 13.42% (7.16–19.68), 7.97% (3.56–12.38), and 3.72% (1.75–5.69) in An Giang province, Ho Chi Minh City, Dak Lak province, and Hue City respectively. However, the age-stratified seroprevalence suggests that the last transmission ended around 30 years ago, consistent with results from the systematic review. We see no evidence for on-going transmission in three of the locations, though with some evidence of recent exposure in Dak Lak, most likely due to transmission in neighbouring countries. Before the 1980s, when transmission was occurring, we estimate on average 2–4% of the population were infected each year in HCMC and An Giang and Hue (though transmision ended earlier in Hue). We estimate lower transmission in Dak Lak, with around 1% of the population infected each year.

**Conclusion:**

In conclusion, we find evidence of past CHIKV transmission in central and southern Vietnam, but no evidence of recent sustained transmission. When transmission of CHIKV did occur, it appeared to be widespread and affect a geographically diverse population. The estimated susceptibility of the population to chikungunya is continually increasing, therefore the possibility of future CHIKV transmission in Vietnam remains.

## Author summary

In recent years, the reemergence of chikungunya has gained global attention and threatened to become a global outbreak. Although the epidemiology of chikungunya is known globally, the viral activity in Vietnam has not been thoroughly investigated. In this paper, we used information from a systematic review and serological survey to understand the past activity of chikungunya in Vietnam. In the serological survey, we tested age -stratified population serum samples in the central and southern parts of Vietnam from 2015 for chikungunya IgG, which is indicative of past exposure to chikungunya. Our results show almost no positive individuals below the age of 30 years old, indicating very low to no virus transmission for the last 30 years. In individuals over 30 years old, there were more positive individuals, and we estimate widespread transmission with an average of 2–4% of the population infected each year. Because of the lack of recent transmission in these locations, the susceptible population is increasing and is now high, suggesting Vietnam is vulnerable to future outbreaks if chikungunya is reintroduced. Hence, chikungunya surveillance along with surveillance for other arboviruses, such as dengue, should be conducted in Vietnam.

## Introduction

Chikungunya virus (CHIKV) belongs to alphavirus of the family Togaviridae. The name chikungunya is derived from the East African language Makonde from the root verb kungunyala which means “that which bends up”, describing the stooped posture in CHIKV cases caused by swelling, stiff joints, and muscle pains. The most prominent symptom of CHIKV infection is high fever and joint pain in the acute phase. Symptoms include headache, diffuse back pain, myalgia, nausea, vomiting, polyarthritis, rash, and conjunctivitis. Due to the similarity of symptoms, CHIKV can be misdiagnosed as dengue fever, especially in the acute phase. The laboratory tests to differentiate dengue virus (DENV) from CHIKV infection only work days after symptom onset and are not commonly recommended, though an algorithm for testing for Zika virus (ZIKV), CHIKV, and DENV has recently been published by CDC [1]. For some individuals, rheumatic symptoms can occur 2–3 months after the acute phase [2, 3]. The primary vectors are the *Aedes aegypti* and *Aedes albopictus* mosquitoes. These vectors also spread DENV, which explains why the distributions of these two viruses overlap. Once infected, as far as is known, antibody may last for life and individuals will be protected against reinfection for a long time [4]. The proportion of infections that are asymptomatic varies widely and has been reported to be 3.2%, 18%, or even 82.1% [5–7].

The first clinical report of CHIKV fever was as early as the 1770s [8] and the first serological test of CHIKV infection was validated after an epidemic in Tanzania in 1952–1953 [9]. After the Tanzania outbreak, the virus began to be detected throughout sub-Saharan Africa, India, and countries in Southeast Asia, leading to numerous epidemic reports in subsequent years [10]. The first outbreak in Asia was reported from Bangkok in 1958 [11] and was thought to lead to the initial epidemics in Cambodia, Vietnam, Malaysia, and Taiwan. In a 2005–2006 outbreak on La Re'union Island in the Indian Ocean, CHIKV started to present with very complicated manifestations, primarily associated with encephalopathy and hemorrhagic fever [12, 13]. This epidemic also marked the first CHIKV deaths, as well as the first cases of peripartum mother-to-child transmission [14–16]. Particularly significant was the extent of this outbreak, with 220,000 people, nearly 40% of the population, infected. The second recent CHIKV reemergence caused outbreaks in Malaysia (2006) and various Pacific islands with ongoing circulation since 2011 [17–19]. In 2013, CHIKV transmission was detected in St. Martin in the Caribbean [20], later spreading to 45 countries and territories in the Americas. The outbreaks in the Americas caused about 1.1 million cases in a year [21]. From December 2013 to March 2017, there have been over 2.4 million cases reported, including severe cases and deaths [22].

Little is known about CHIKV transmission in Vietnam, where dengue is endemic and *Aedes* mosquitoes are abundant. Since CHIKV is easily misdiagnosed as DENV because of the similar symptoms, it is possible that CHIKV transmission in Vietnam could be masked by ongoing DENV transmission. Previous studies have used serology to study CHIKV activity in Brazil, India, Caribbean islands, the Union of Comoros and Tanzania [23–27]. In order to test for past exposure of infections in the population, IgG ELISA test is often used as it is commercially available and relatively easy to perfom [26–30]. These studies can provide information about the extent of present and past transmission and can help us quantify parameters of transmission. In this study, we investigated the past and current transmission of CHIKV in Vietnam by undertaking a systematic review and performing a population seroprevalence survey using IgG ELISA at four locations across the country. With the results of the serosurvey we used a mathematical model to infer parameters of transmission in the four locations across Vietnam.

## Methods

### Ethics statement

The Scientific and Ethical Committee of the Hospital for Tropical Diseases in Ho Chi Minh City and the Oxford Tropical Research Ethics Committee at the University of Oxford approved the study.

### Systematic review

We performed a systematic review by searching for information in international journals, national Vietnamese journals, and online news. The key word “chikungunya Vietnam” was used in the search engines: PubMed, Web of Science, ProMED and Google news search. For the Google news search engine, the search results only from Vietnam in the period 01/01/2000 to 03/21/2017 were used.

The key word “chikungunya” was used in local search engines for papers and theses: Vietnam Journal of Preventive Medicine—a peer-reviewed journal in Vietnam [31] and a National Library of Vietnam–Ph.D. thesis storage, which contains electronic versions of about 21.300 Ph.D. theses (full-texts or summaries) [32].

### Seroprevalence study

**Fig 1:**
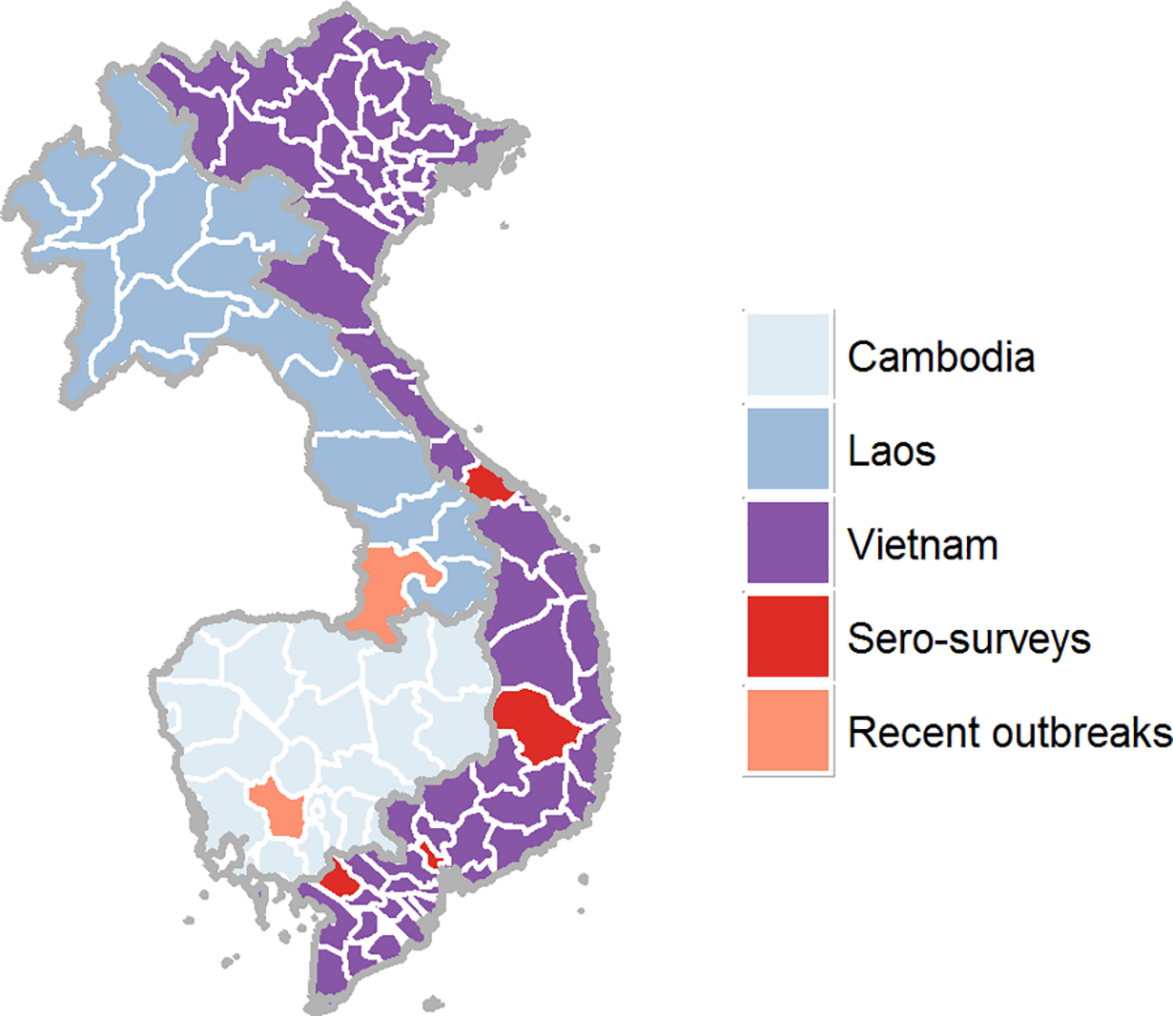
Locations of serum sample collection and recent outbreaks locations: From north to south, Hue City, Dak Lak province, Ho Chi Minh City, and An Giang province are shown in red. Locations with recent outbreaks are shown in orange; Kampong Speu province in Cambodia (2012) and Champasak province in Laos (2012). The map was generated in R software version 3.3.3 [37].

As part of a large ongoing study of serial seroepidemiology, residual serum samples are collected from four hospital laboratories in southern Vietnam: the Hospital for Tropical Diseases in Ho Chi Minh City, An Giang General Hospital, Dak Lak General Hospital, and Hue Central Hospital [33–36]. For this study we selected age stratified samples from those collected in 2015. Geographic locations are shown in Fig 1. The patients came to those hospitals as inpatients or outpatients; admitting ward for inpatients is recorded, but diagnosis and reason for visit are not. All samples were anonymized.

In each location, we tested 136 or 137 samples and aimed for at least 15 samples in each of the following age groups: 1–10 years, 11–20, 21–30, 31–40, 41–50, 51–60, 61–70, 71+ years. In some of the older age groups, there were fewer than 15 samples in the serum bank. However, each age group contained at least 10 samples. The serum samples were tested for the presence of CHIKV IgG antibody using the ELISA kit for CHIKV–NovaLisa [38, 39]. The absorbance values of the samples were translated to NovaTec Units (NTU). According to the manufacturer's guidelines, samples were defined as positive with > 11 NTU, negative with <9 NTU, and inconclusive with 9–11 NTU.

We assessed whether there were any differences in proportion positive by gender, sample locations, and original admission wards. The binomial 95% confidence intervals for each age group were calculated using the Wilson score interval method. We also calculate the age-adjusted seropositivity for each location using the direct standardization method with the Vietnam standard population from the World Population: the 2015 Revision online dataset, conducted by the United Nations [40]. All analyses were performed in R software version 3.3.3 [37].

### Force of infection estimation

The force of infection (FOI) is defined as the per capita rate at which susceptible individuals are infected by an infectious disease. In this study, we used Muench’s catalytic model [41] to estimate the FOI varying in time but not with age. The ELISA results of seropositives and seronegatives were stratified into 15 age groups, each spanning five years. The likelihood of a single positive individual in age group *n* is:

$$L_{n}^{pos}=1-exp(-5\sum_{i=1}^{n-1}\lambda_{i}-2.5\lambda_{n})$$ (1)

The likelihood of a single negative individual in age group *n* is:

$$L_{n}^{neg}=exp(-5\sum_{i=1}^{n-1}\lambda_{i}-2.5\lambda_{n})$$ (2)

With *λ*_*i*_ (*i* = 1,2,…, 14) the average annual FOI in respective period 2011–2015, 2006–2010, 2001–2005 through to 1946–1950. *λ*_15_ represents for the annual FOI estimated for the period 1931–1945. Further details of the annual FOI estimation from 1931 to 2015 can be found in S1 Text. We introduce index *i*_*end*_ to the model to infer the last time period that transmission occurred. The index is defined so that every FOI before it (which means FOI values of more recent years) are fixed at near 0. For other time periods, a flat uninformative prior was used. The model fit was run for each index value and the 16 different models were compared using the DIC values. We fit the model using RStan package [42]. Further details of this model can be found in S1 Text.

### The susceptible proportion in each year period

From the average annual FOI, we can derive the susceptible proportion over time. Our model assumes that the chance of being seropositive results only from transmission in that location, and that there was no inward migration or individuals acquiring infection elsewhere. From 1930 to 2015, the proportions of susceptible individuals in each age group were calculated for each iteration of the model by a formula:

$$S_{j}=\sum_{k=0}^{80+}a_{j,k}e^{-\lambda_{j,k}^{aff}}$$ (3)

*S*_*j*_ is the proportion of the susceptibles in each year *j* = 1930, 1931,…, 2015. *a*_*j*,*k*_ is the age-specific proportion of year *j* for each age group *k* = 0,1,2,…, 79,80 +. For the time periods before we are able to estimate FOI from our data, we consider two scenarios. The “no endemic scenario” hypothesizes there was no transmission prior to 1930, while the “endemic scenario” takes the FOI prior to 1930 as equal to the FOI estimated for 1931. $\lambda_{j,k}^{aff}$ is the FOI that affects the age group *k* in the population in year *j*. Full descriptions of $\lambda_{j,k}^{aff}$ definition with the 2 endemic scenarios are in S1 Text.

From 1950 to 2015, the demographic data comes from the World Population Prospects: the 2015 Revision online dataset, conducted by the United Nations as above [40]. Another paper provided the estimated age composition data of 1929–1944 every 5 years [43]. The years in between were inferred using linear interpolation.

## Results

### Systematic review

**Fig 2:**
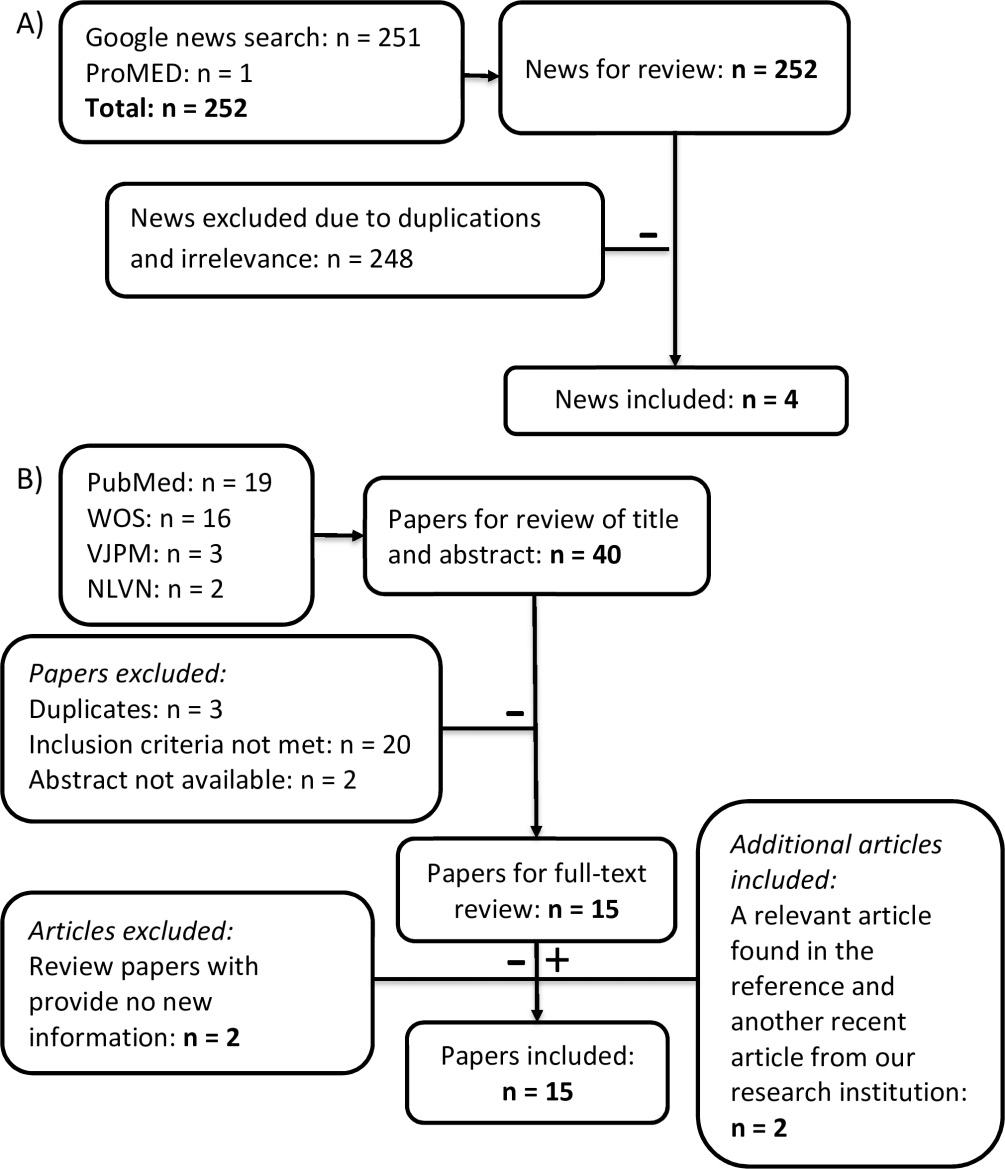
Flowchart describing the systematic review procedure searching for information of CHIKV activity in Vietnam. **A) Searching procedure in Google news and ProMED. B) Searching procedure in international journals and national Vietnamese journals**. Abbreviations: WOS = Web of Science, VJPM = Vietnam Journal of Preventive Medicine, and NLVN = National Library of Vietnam.

Forty papers were found in different search engines and reviewed (see Fig 2). Further details of all abstracts are in S1 Table. One paper [44] was in PubMed but it was not possible to find the abstract. There were 3 duplicates between PubMed and Web of Science. After further ruling out 20 irrelevant abstracts and 2 unavailable abstracts, the full-text of 15 abstracts were inspected. There are two references with information on fever of unknown origin in United States soldiers in Vietnam [45, 46], which was already thoroughly recorded in another paper [47]. In the process of full-text reviewing, one more relevant Vietnamese paper [48] was found in the reference of another paper [49]. We also include a recent study of CHIKV from our research institution [50]. Finally, 15 papers were included.

There were 251 articles from Google News search and one report from ProMED search. Most articles mentioned CHIKV only briefly in the context of recent ZIKV management in Vietnam. There were only 3 relevant unique articles from Google News and a ProMED report included.

From the systematic searches we were able to assemble information about the past and current transmission of CHIKV in Vietnam. In August 1963, there was an outbreak of hemorrhagic fever disease in children in the Mekong River Delta (southern Vietnam) [51], characterized by shock and a high mortality rate [52, 53]. Research on this outbreak showed that several children from An Giang province (rural area) and Ho Chi Minh city (urban area) with acute hemorrhagic fever had evidence of CHIKV infection (high hemagglutination-inhibition (HI) and complement-fixation (CF) antibodies titres to CHIKV) [51]. In that same study, 22 of 75 serum samples from healthy children in Ho Chi Minh City had detectable CHIKV HI antibody, suggesting past CHIKV transmission in Ho Chi Minh City [51]. Serosurveys on a larger scale from consecutive years (1963–1965) also reported the proportion of seropositive people in Ho Chi Minh City as 31.36% by CF in 1963 and 10.26% by HI in 1964–65 [54, 55].

In the following years, there were scattered reports of CHIKV from 1963 to 1982. In summary, in 1966, 10 out of 110 American soldiers with fever of unknown origin in southern Vietnam tested positive by virus isolation to CHIKV [47]. In 1972, in a large serosurvey in three locations in southern Vietnam, 21 out of 130 suspected cases of DENV had positive CHIKV PRNT [56]. Finally, a multi-year study reporting hemorrhagic fever cases in southern Vietnam, found 12 cases with positive CHIKV virus isolation from 1978 to 1982 [57]. From 1983 to 1986, all tests in this survey were negative and after 1987 the survey was stopped. This suggests transmission of CHIKV may have ended in 1982. There were no papers reporting testing for CHIKV between 1986 and 2005.

More recently, a retrospective study of samples from febrile individuals in 6 countries in South East Asia and Fiji from 2005 to 2006 found IgM antibody positive cases of CHIKV in some countries but none in Vietnam. However, 11 of 44 samples from febrile patients tested in Hanoi (northern Vietnam) were positive by IgG ELISA and confirmed by neutralization assay [58]. In 2009, there was a 14-fold increase in cases with dengue-like symptoms compared to previous years and 60 percent of patients with classic dengue-like symptoms tested negative for DENV [59]. Of the acute hemorrhagic fever patients tested, 4/50 were PCR positive to CHIKV and genetic sequencing showed > 93% similarity with the CHIKV derived from Africa (S27 subtype) [48]. In 2010, the National Institute of Hygiene and Epidemiology (NIHE) announced 15 patients with hemorrhagic fever who were negative for DENV and positive to CHIKV [60]. From September 2010 to June 2011, a cohort study conducted in My Tho city in southern Vietnam reported that a total of 19 cases out of 32 febrile children tested were positive by ELISA IgM CHIKV [61, 62]. After 2011, due to the CHIKV outbreaks in Laos and Cambodia [63, 64], several studies were conducted in the border locations in Vietnam but showed no activity of the virus [49, 65–67]. A recent study tested 8015 febrile children in southern Vietnam and revealed four positive cases of CHIKV by RT-PCR [50]. The positive samples were found between August and November 2012, with one case from Ho Chi Minh City and three from Binh Duong province (about 20 kilometers away from the Cambodian border). Phylogenetic analysis indicated those four strains were closely related to the Cambodia strain which caused the outbreaks in 2012 [63].

In early 2016, a news report stated that of 83 people with Zika-like symptoms tested across eight provinces in Vietnam, nine individuals from Can Tho City tested positive to CHIKV[65]. However, the test used was not reported. In April 2016, NIHE found 56 (0.29%) *A*. *aegypti* that were positive to ZIKV, 29 (0.12%) positive to DENV but none positive to CHIKV [68].

### Seroprevalence study

**Fig 3:**
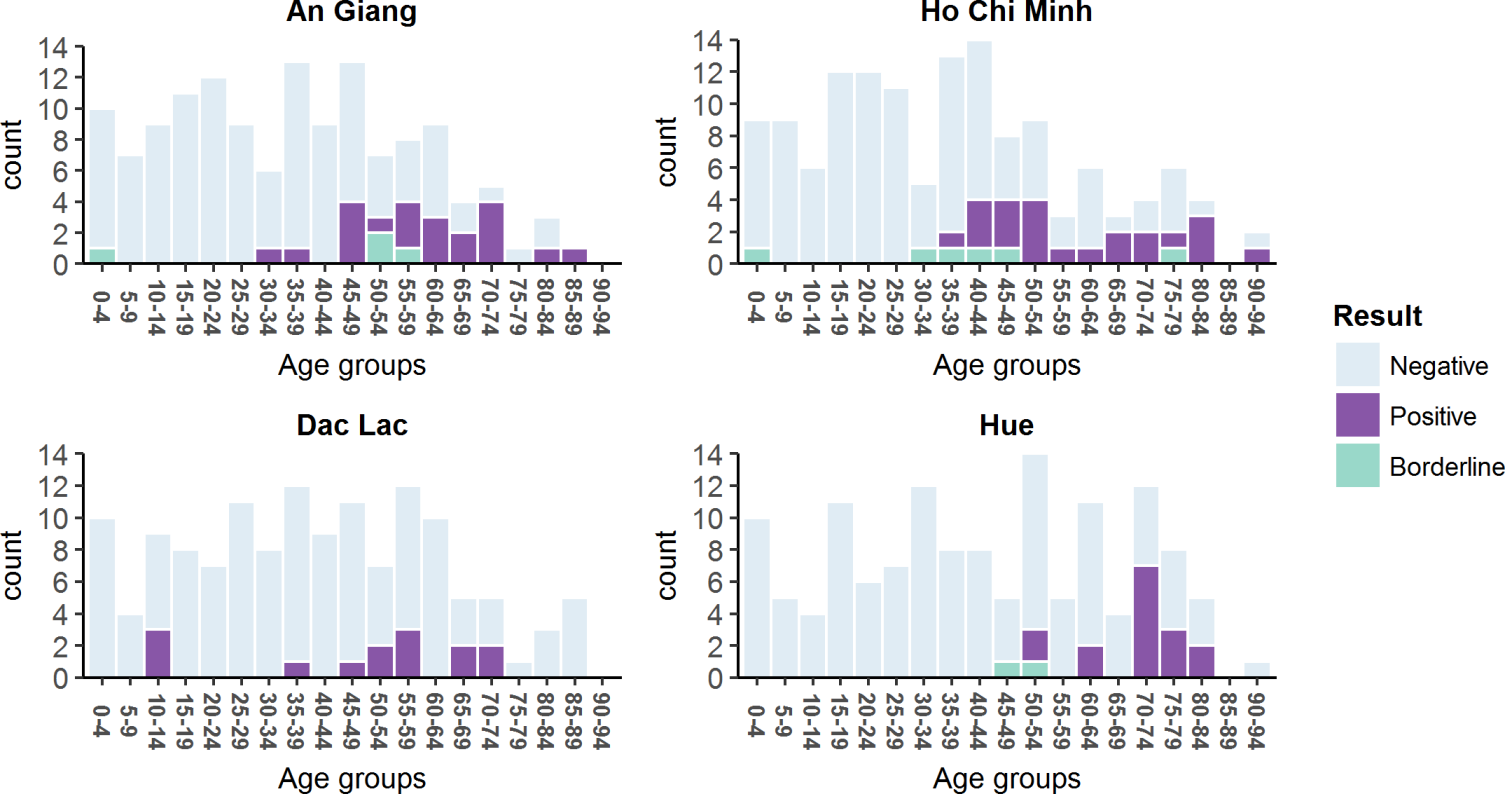
The stacked barplots of the numbers of negative, positive, borderline ELISA results by age group in each location: An Giang province, Ho Chi Minh City, Dak Lak province, and Hue City. The colored bars represent the negative, positive, and borderline respectively (as shown in the legend).

In this study, 546 individuals were tested for CHIKV IgG using the NovaTec CHIKV ELISA. Individuals were defined as positive, negative, or borderline depending on the NovaTec titres as defined in the methods. The full dataset is in S1 Data. Across all ages, there were 21, 22, 16, 14 positive individuals in An Giang, Ho Chi Minh, Dak Lak, and Hue respectively. Fig 3 shows the numbers of positive, negative and borderline cases by 5 year age group for each location (more detail in S2 Table).

The direct age-adjusted seropositivity percentages were 12.30% (6.58–18.02), 13.42% (7.16–19.68), 7.97% (3.56–12.38), and 3.72% (1.75–5.69) for An Giang province, Ho Chi Minh City, Dak Lak province, and Hue City respectively. For males the percentage positive was 18% (9.8–30.8), 19.6% (11.0–32.5), 10.2% (6.2–16.4), and 14.5% (7.6–26.2); and for females they were 14.5% (8.5–23.6), 15.2% (8.9–24.7), 10.2% (4.7–20.5), and 10.1% (5.2–18.7), for each province respectively. There were no statistical differences between the proportion positive in males and females. Samples in this survey come from people admitted to different hospital wards and we saw no difference in positivity by ward of admission (full details in the S2 Table).

**Fig 4:**
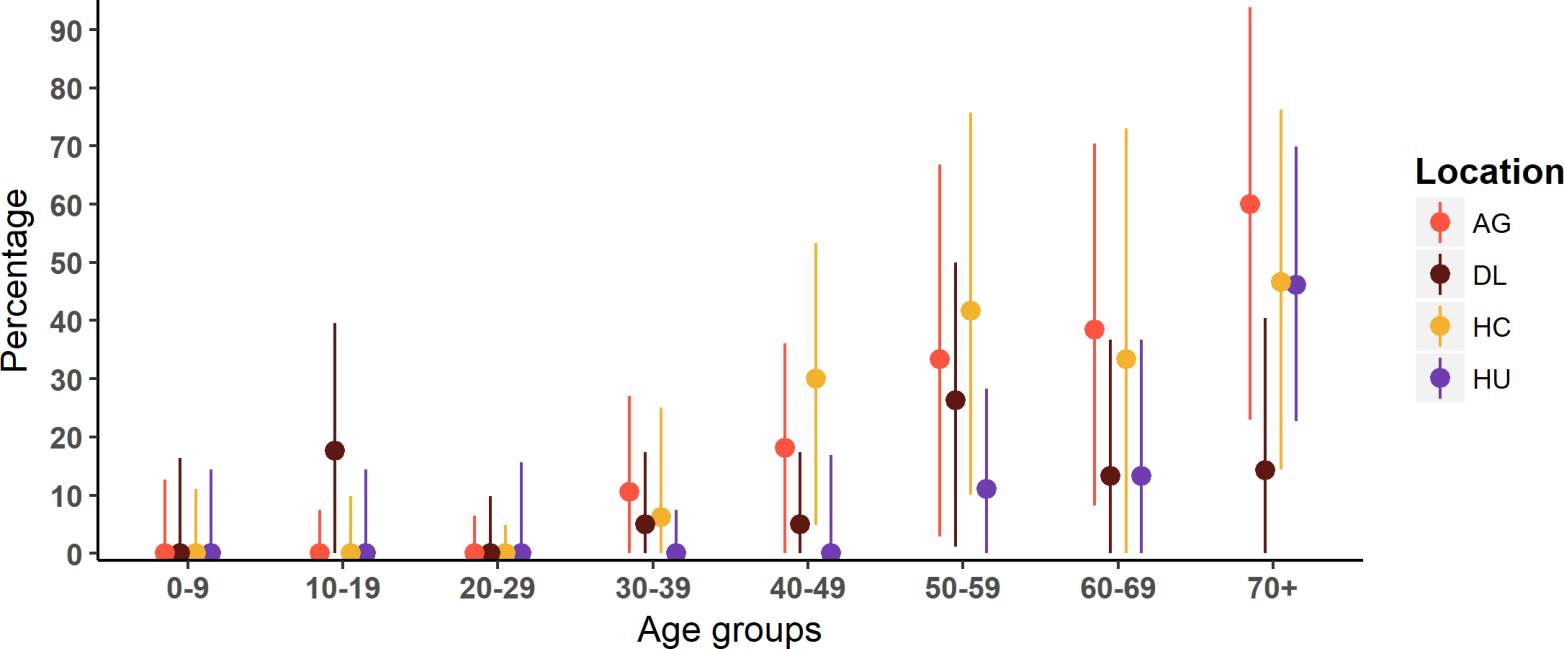
Percentage of positive individuals and the binomial proportion confidence intervals by age group in each location: The binomial 95% confidence intervals are corrected by known 90% sensitivity and 90% specificity of the diagnosis test [69]. The colors represent each location, with red for An Giang province, brown for Dak Lak province, orange for Ho Chi Minh City, and purple for Hue City.

It is noteworthy that almost all the positive tests were in those 30 years old and above, except for Dak Lak where three children were positive (two 10-year-olds and one 13-year-old, more detail in S3 Table). The proportion of positive individuals and binomial 95% confidence intervals by age groups (excluding borderline results) are shown in Fig 4.

### Model fit and force of infection estimates

The DIC of the different models with transmission ending in each of the 5 year periods are shown in Table 1. In An Giang, the best model (smallest DIC) is with transmission ending in 1986–1990. In Ho Chi Minh City, the model ranking supports endemic transmission ending in 1976–1980. The transmission in Dak Lak is estimated to be more recent and Hue, on the other hand, is estimated to have the longest time without CHIKV transmission—since 1966–1970. There are large changes in DIC values for each location, hence clearly suggesting transmission before 1980, 1975, 2000, and 1960 in An Giang, Ho Chi Minh, Dak Lak, and Hue respectively. Based on [70, 71], if we choose models that have DIC differences < 3 compared to the best model, we estimate that transmission ended in the periods 1981–2000, 1976–1995, 2001–2015, and 1961–1980 in An Giang, Ho Chi Minh, Dak Lak, and Hue respectively. Though (with a DIC difference between 3 and 5) we cannot rule out slightly more recent transmission in An Giang, Ho Chi Minh, and Hue, the DIC values clearly suggest no on-going transmission in these locations. FOI estimates and model fits for 3 best models in each location shown in Figure A and B in S2 Text.

**Table 1:** Table of DIC values calculated from 16 models in 4 locations. Each model is defined to have different times of transmission ending, as shown in the first column of the table. Yellow coloured cells are the best models in each location. Green colored cells are the models which have DIC value rank 2^nd^ and 3^rd^ from the bottom up, hence the 2^nd^ and 3^rd^ best models. The FOI estimates from the 2^nd^ and 3^rd^ models are shown in Figure A in S2 Text.

Time period	An Giang	Ho Chi Minh	Dak Lak	Hue
2011–2015	92.38	96.08	100.05	79.50
2006–2010	91.18	94.76	100.56	78.01
2001–2005	89.71	93.06	102.90	76.45
1996–2000	88.55	91.62	181.69	74.78
1991–1995	87.15	90.10	180.50	73.19
1986–1990	86.48	88.82	179.57	71.75
1981–1985	86.65	87.90	178.72	70.28
1976–1980	114.19	87.62	178.84	68.91
1971–1975	139.64	115.92	202.78	67.83
1966–1970	139.46	203.97	202.85	67.23
1961–1965	253.52	292.02	229.92	67.72
1956–1960	281.77	403.14	285.48	121.05
1951–1955	365.75	428.80	360.89	120.95
1946–1950	448.20	455.47	360.79	173.49
1931–1945	503.34	510.81	412.43	172.58
Before 1930	672.27	702.77	461.70	514.09

**Fig 5:**
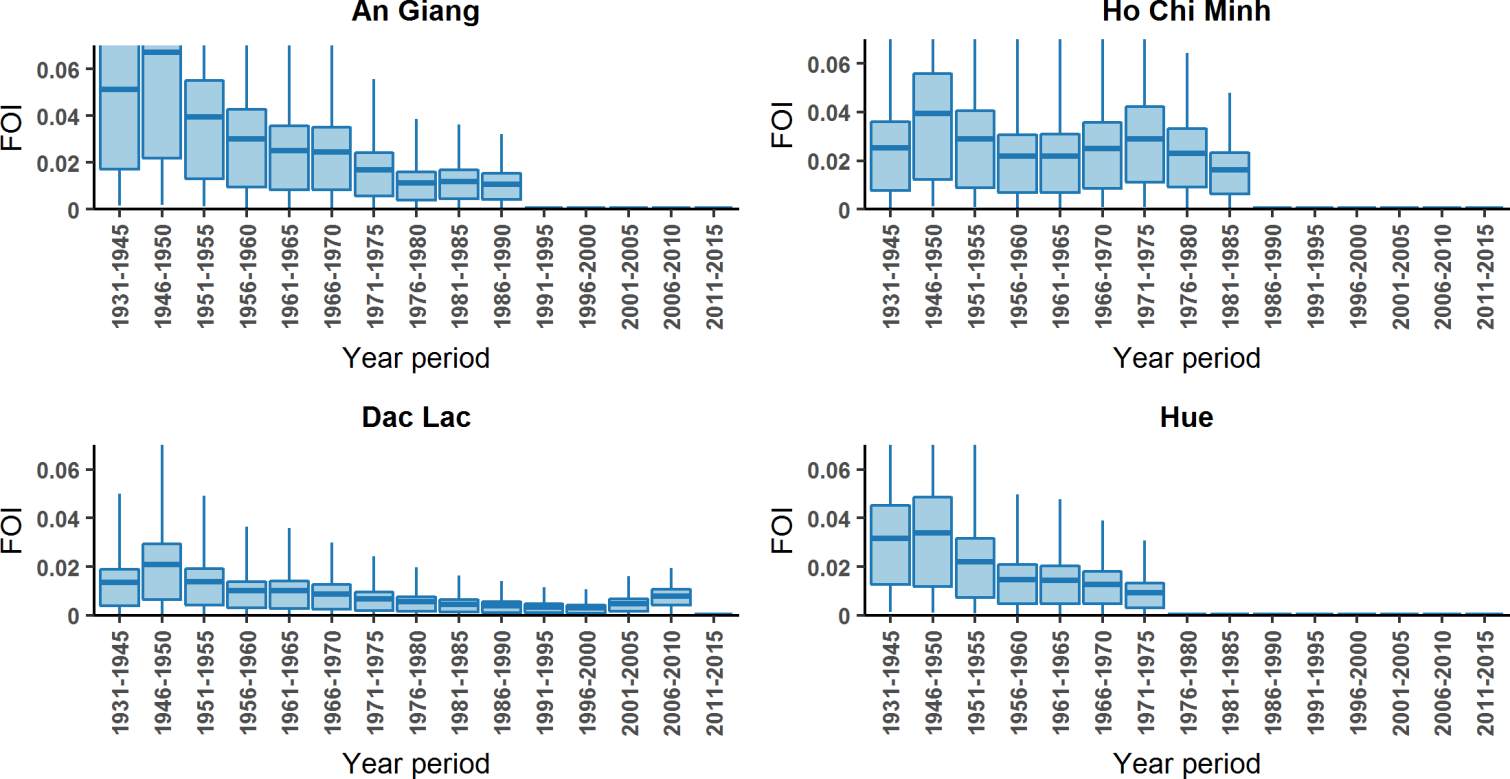
FOI estimates in each period by location from the best fit model. The blue boxplots show the credible intervals with means, 1^st^ quartile and 3^rd^ quartile of annual FOI estimation by time period in each location.

**Fig 6:**
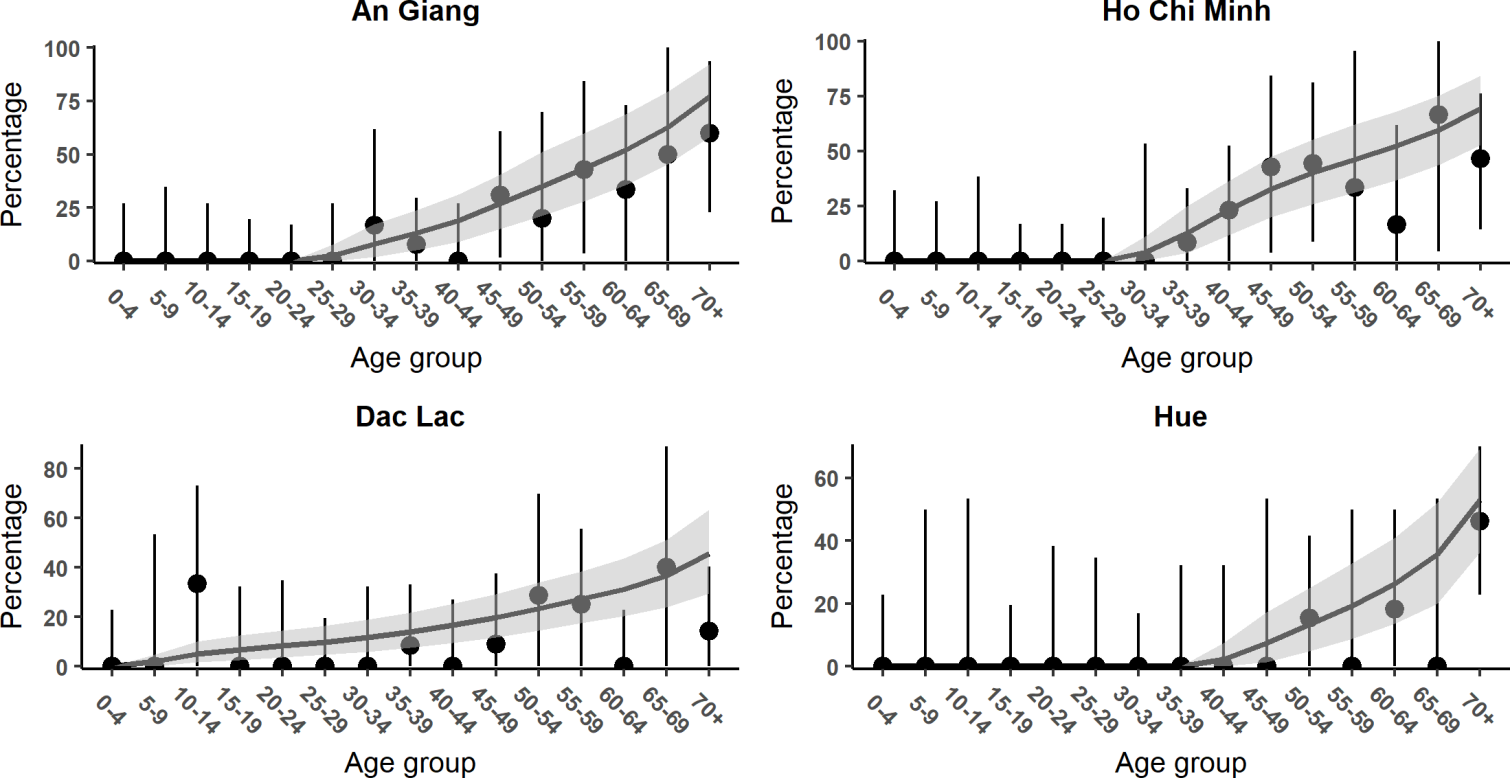
Model fit of the best fitting model to the age-specific seropositive proportion in each location. The black dots are the seropositive proportion in each age group along with corresponding binomial 95% confidence intervals, corrected by reported 90% sensitivity and 90% specificity of the test [69]. The black lines are the model output generated from the model simulated with the mean parameters estimates. The grey areas represent the 95% credible intervals.

Annual FOI estimates by time period from the model with the smallest DIC value for each location are shown in Fig 5 (full posterior distributions shown in Figure A in S1 Text). We estimate An Giang to have the highest transmission in 1931 to 1955 with a peak of FOI of 0.067 (0.001–0.222) in 1946–1950. In Ho Chi Minh City there is a similar peak in transmission estimated in 1946–1950 of 0.040 (0.001–0.136), but transmission is estimated to be higher than An Giang in the later years from 1966 to 1980. In Dak Lak, we estimate low transmission with almost no mean FOI estimated larger than 0.02 in any period). The peak of FOI in 2006–2010 hints a recent activity of CHIKV in this area though the model does not capture the rest of the data well. In Hue, the magnitudes of the past FOI are comparable to Ho Chi Minh City. The model output and the seroprevalence data for each location is shown in Fig 6.

We estimated the FOI as an average over five year periods. In order to assess how sensitive our results were to this assumption we estimated the FOI for models with 1–5 year groups (S1 Text). The results suggest that our data may not have enough resolution for FOI estimation in 1–2 years however the 5 year group models fit similarly to the 3–4 year group models and estimate transmission ending at broadly similar times for all locations.

**Fig 7:**
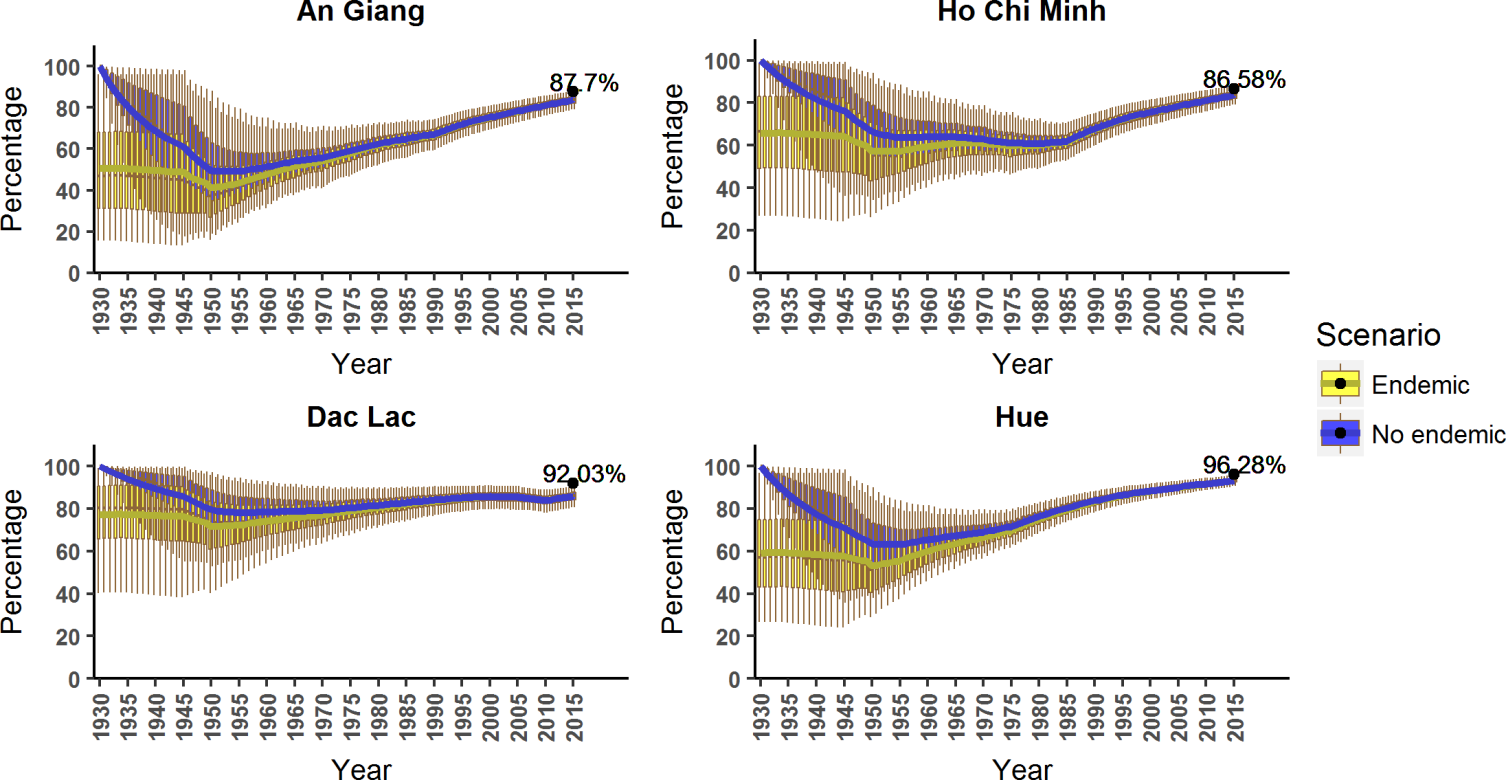
The predicted susceptible proportions over time from the best model in each location. In all locations, the boxplots represent the generated proportion with its 95% credible intervals (also shown 1^st^ quartile, 3^rd^ quartile, and the medians) with the solid lines showing the mean value of each interval. The two scenarios, with or without endemic transmission before in 1930 are shown in the plot by yellow and blue respectively. The black dots are the age-adjusted seropositive proportions from our serosurvey in 2015.

Based on the FOI estimates, we were able to infer changes in the susceptible proportion over time as shown in Fig 7. Depending on our assumption about previous transmission before 1930, there are differences in our estimates of the susceptible proportion in the early years. However, the discrepancy becomes negligible in later years. The higher FOI in Hue, HCMC and An Giang means we estimate the greatest decrease in the susceptible proportions in these locations. In Dak Lak, by contrast, the susceptible proportion remains high at all times. These results are consistent across the top three models for all locations (see Figure C and D in S2 Text). Due to the estimated lack of transmission in recent years, we see a steady increase in the susceptible proportion in recent years, up to over 85% in all locations in 2015.

## Discussion

Using two complementary methods, a systematic review and a seroprevalence study, we have been able to provide a description of past and present CHIKV transmission in central and southern Vietnam. We conclude that there has been little to no recent transmission in these locations in Vietnam, but that CHIV was last circulating widely in the early 1980s.

In the locations where there was past literature on CHIKV cases (HCMC and An Giang) results provided solid evidence of CHIKV transmission in the periods 1963–1966 and 1978–1982. The results from the force of infection estimates, estimate transmission in the intervening years (with increases in the proportion seropositive in the relevant age groups leading to estimates of transmission in these time periods (Fig 5)). Therefore we conclude consistent transmission before 1980s, though cannot rule out multiple smaller outbreaks rather than one large outbreak.

With the serological results, we were able to reconstruct the magnitude of CHIKV transmission that occurred before the 1980s. In two of the areas we surveyed, HCMC and An Giang, we estimated the highest and most consistent transmission, with an average of 2–4% of the population being infected each year. The results from previous serosurveys in 1963–1965 estimated between 10 and 30% seropositivity depending on the methods used [54, 55]. For the same time period we estimated between 10 and 40% (depending on the scenario used for past transmission), which is consistent with this estimate.

In Dak Lak, three seropositive individuals less than 30 years old caused the estimates of the FOI in recent years to be slightly greater than 0. As the model does not incorporate external introductions, we cannot determine if these seropositive individuals were caused by introductions or by low level local transmission. Supporting the external introduction theory, Dak Lak borders Cambodia and there could be limited transmission from Cambodia into Dak Lak province; alternatively, some of those found to be positive could have traveled to Cambodia and been infected there. In the systematic review, we did not find any information about past transmission in Dak Lak. Hue had the lowest transmission, and Hue’s transmission appeared to end earlier than in Dak Lak, An Giang, or HCMC. We found no information about testing for historical transmission in Hue, however, in a study from 2012 to 2014, no CHIKV activity was found in this location [66].

We can consider the differences we estimate in chikungunya transmission intensity across locations in the context of climate and transmission patterns of DENV, as they are transmitted by the same mosquitoes. An Giang and HCMC in the south are generally hot and have ongoing DENV transmission throughout the year, but with higher numbers of DENV cases in the wet season [39, 72]. The DENV transmission intensity is thought to be higher in these two locations than in the other two we tested [39]. Dak Lak, by contrast, is cooler and has generally lower levels of DENV transmission, however with some years with large numbers of cases. Hue, farther north, has clearer seasons and generally lower DENV case numbers. These patterns across the areas in DENV transmission are broadly consistent with the forces of infections and length of transmission we observed for CHIKV in our study. It is worth noting however that although the same mosquitoes can transmit CHIKV and DENV, the way climate impacts the virus transmission may differ [73].

It is useful to compare our findings on force of infection and the proportion seropositive after CHIKV outbreaks to those in other settings. A recent CHIKV seroprevalence study in Chennai, India found steady consistent seropositive proportions across 5–40 years old, suggesting epidemic but not endemic transmission [29]. Our results are comparable to those in Cebu in Philippines which estimated the susceptible population to be above 50% at all times despite large outbreaks [70]. It would be interesting to see estimates of the FOI and the proportion that remain susceptible after the recent outbreaks in South and Central America.

There are limitations to our study. Our samples are from individuals who have attended hospital for some reason, so are not fully representative of the population. However, for the younger individuals, we would assume this would bias the study towards finding positive individuals as in Berto et al [35], which we did not. For older individuals, this may be more representative of the population as a whole and as the exposure is assumed to be over 30 years ago, it is reasonable to assume this hospital visit is unrelated to the CHIKV infection exposure. It is also reasonable to assume that CHIKV did not lead to death in a significant proportion of individuals [10], therefore, our seroprevalence estimates are unbiased. We did not have samples from Hanoi, despite reports of recent chikungunya transmission there. Future serological studies in Hanoi would be of interest.

The three hospitals in An Giang, Dak Lak, and Hue province, are the largest hospitals in the province and therefore are likely to geographically represent the population in the respective areas. However, for the hospital in HCMC, patients may come from across the south of Vietnam and not just from the city. To assess this possible bias, we presented analysis for HCMC and An Giang together in S1 Text, and the findings are not different from the results for each location. In addition, by stratifying the serological results to 5-age groups, our model might lose some finer details from the exact age of each individual; however we show that the trend of the results is consistent across models with different age groupings.

Another limitation with any serological survey is concern about cross-reactivity. The manufacturers state that the test has no cross-reactivity to DENV virus, tick-borne encephalitis, CMV, EBV and Helicobacter pylori and that the diagnostic specificity and sensitivity are > 90% [38]. Cross-reactivity with O'Nyong Nyong virus is not excluded, however, there are no reports of O’Nyong Nyong virus in Vietnam, and the virus is thought to be limited geographically to East Africa [74–76]. However we cannot rule out cross-reactivity with other unknown alphaviruses.

In summary, through a systematic review and seroprevalence study, we have provided information on the past and current transmission of CHIKV in southern and central Vietnam. We saw evidence of widespread transmission of CHIKV over 30 years ago, with variation across locations. It is not clear why transmission ended, but there may be lessons here for what we could expect to occur in South and Central America after a number of years of widespread transmission. We estimate that there is high and increasing susceptibility to CHIKV in southern and central Vietnam suggesting that the population is vulnerable to a CHIKV outbreak should the virus be re-introduced to Vietnam, therefore public health agencies should be vigilant for chikungunya cases.

## Supporting information

S1 Checklist STROBE checklist. (PDF)

S2 Checklist PRISMA checklist. (PDF)

S1 Flowchart PRISMA 2009 flowchart. (PDF)

S1 Text Supplement text of evidence of previous transmission of CHIKV in Vietnam. (DOCX)

S2 Text Comparisons of FOI estimation, susceptible proportion of best models. (DOCX)

S1 Table Description of papers included in literature results. (DOCX)

S2 Table The summarized results table of negative, borderline, positive, and total cases by total data, sex, ward at admission, also the age demographic (Minimum, maximum, mean, median, and standard deviation values) for each location. (DOCX)

S3 Table Demographics of 3 positive children from Dak Lak province. (DOCX)

S1 Data The CHIKV dataset in 4 locations in Vietnam. (XLSX)
