# Supplementary material for: Evidence of previous but not current transmission of chikungunya virus in southern and central Vietnam: Results from a systematic review and a seroprevalence study in four locations
Source: PLoS Negl Trop Dis. 2018 Feb 9;12(2):e0006246. doi: 10.1371/journal.pntd.0006246 (PMC5823466; doi:10.1371/journal.pntd.0006246)
Supplement: S1 Checklist — (PDF) [file pntd.0006246.s001.pdf]

STROBE Statement—checklist of items that should be included in reports of observational studies

	Item No.	Recommendation	Page No.	Relevant text from manuscript
**Title and abstract**	1	(*a*) Indicate the study's design with a commonly used term in the title or the abstract	1	Results from a systematic review and a seroprevalence study in four locations
		(*b*) Provide in the abstract an informative and balanced summary of what was done and what was found	1,2	What was done: In order to address this question … four locations in Vietnam<br>What was found: The findings give us evidence of some CHIKV activity … 1% of the population infected each year.
**Introduction**				
Background/rationale	2	Explain the scientific background and rationale for the investigation being reported	2,3	Scientific background:<br>Chikungunya virus (CHIKV) belongs to alphavirus of the family Togaviridae ... Through March 2017, there have been over 2.4 million cases reported, including severe cases and deaths [21].<br><br>Rationale:<br>Little is known about CHIKV transmission in Vietnam, where dengue is endemic and Aedes mosquitoes are abundant … These studies can provide information about the extent of

				present and past transmission and can help us quantify parameters of transmission.
Objectives	3	State specific objectives, including any prespecified hypotheses	3	In this study, we investigated the past and current transmission of CHIKV … we used a mathematical model to infer parameters of transmission in the four locations in Vietnam
**Methods**				
Study design	4	Present key elements of study design early in the paper	3,4	Systematic review: We performed a literature review by searching for information in international journals, national Vietnamese journals, and online news.<br>Seroprevalence study: As part of a large ongoing study of serial seroepidemiology, serum samples are collected
Setting	5	Describe the setting, locations, and relevant dates, including periods of recruitment, exposure, follow-up, and data collection	4	As part of a large ongoing study of serial seroepidemiology … All samples were anonymized.
Participants	6	(*a*) *Cohort study*—Give the eligibility criteria, and the sources and methods of selection of participants. Describe methods of follow-up<br>*Case-control study*—Give the eligibility criteria, and the sources and methods of case ascertainment and control selection. Give the rationale for the choice of cases and controls<br>*Cross-sectional study*—Give the eligibility criteria, and the sources and methods of selection of participants	4	As part of a large ongoing study of serial seroepidemiology, … but diagnosis and reason for visit are not.
		(*b*) *Cohort study*—For matched studies, give matching criteria and number of exposed and unexposed		

		Case-control study—For matched studies, give matching criteria and the number of controls per case		
Variables	7	Clearly define all outcomes, exposures, predictors, potential confounders, and effect modifiers. Give diagnostic criteria, if applicable	4	In each location, we tested 136 or 137 samples … and inconclusive with 9-11 NTU.
Data sources/ measurement	8*	For each variable of interest, give sources of data and details of methods of assessment (measurement). Describe comparability of assessment methods if there is more than one group	4	In each location, we tested 136 or 137 samples … and inconclusive with 9-11 NTU.
Bias	9	Describe any efforts to address potential sources of bias	4	We assessed whether there were any differences in proportion positive by gender … All analyses were performed in R software version 3.3.3 [38].
Study size	10	Explain how the study size was arrived at	4	In each location, we tested 136 or 137 samples … age group contained at least 10 samples.

Continued on next page

Quantitative variables	11	Explain how quantitative variables were handled in the analyses. If applicable, describe which groupings were chosen and why	4	In each location, we tested 136 or 137 samples and aimed for at least … and inconclusive with 9-11 NTU.
Statistical methods	12	(*a*) Describe all statistical methods, including those used to control for confounding	4,5	We assessed whether there were any differences in proportion positive by gender, … All analyses were performed in R software version 3.3.3 [38]. The force of infection (FOI) … with 2 endemic scenarios are in supplement text.
		(*b*) Describe any methods used to examine subgroups and interactions		Supplement text: section IV. Serosurvey's result summary.
		(*c*) Explain how missing data were addressed		There are no missing data
		(*d*) *Cohort study*—If applicable, explain how loss to follow-up was addressed<br>*Case-control study*—If applicable, explain how matching of cases and controls was addressed<br>*Cross-sectional study*—If applicable, describe analytical methods taking account of sampling strategy	4	In each location, we tested 136 or 137 samples … However, each age group contained at least 10 samples.
		(*e*) Describe any sensitivity analyses		Supplement text: section V. Uncertainty analysis.
**Results**				
Participants	13*	(a) Report numbers of individuals at each stage of study—eg numbers potentially eligible, examined for eligibility, confirmed eligible, included in the study, completing follow-up, and analysed		Not applicable
		(b) Give reasons for non-participation at each stage		Not applicable
		(c) Consider use of a flow diagram		Not applicable
Descriptive data	14*	(a) Give characteristics of study participants (eg demographic, clinical, social) and information on exposures and potential confounders		Supplement text: section IV. Serosurvey's result summary.
		(b) Indicate number of participants with missing data for each variable of interest		There are no missing data
		(c) *Cohort study*—Summarise follow-up time (eg, average and total amount)		
Outcome data	15*	*Cohort study*—Report numbers of outcome events or summary measures over time		

		Case-control study—Report numbers in each exposure category, or summary measures of exposure		
		Cross-sectional study—Report numbers of outcome events or summary measures	,7	Supplement text and also in the main manuscript: In this study, 546 individuals were tested for CHIKV IgG using the NovaTec CHIKV ELISA … (excluding borderline results) are shown in Fig 4.
Main results	16	(*a*) Give unadjusted estimates and, if applicable, confounder-adjusted estimates and their precision (eg, 95% confidence interval). Make clear which confounders were adjusted for and why they were included	7	The direct age-adjusted seropositivity percentages were … for each province respectively.
		(*b*) Report category boundaries when continuous variables were categorized	4	The serum samples were tested … negative with <9 NTU, and inconclusive with 9-11 NTU.
		(*c*) If relevant, consider translating estimates of relative risk into absolute risk for a meaningful time period		Not mentioned

Continued on next page

Other analyses	17	Report other analyses done—eg analyses of subgroups and interactions, and sensitivity analyses	7,8	The log-likelihoods of the different models with transmission … we see a steady increase in the susceptible proportion in recent years, up to over 85% in all locations in 2015.
**Discussion**				
Key results	18	Summarise key results with reference to study objectives	8,9	Using two complementary methods, a literature review and a seroprevalence study… the serological and modelling results were consistent with the results of the literature review.
Limitations	19	Discuss limitations of the study, taking into account sources of potential bias or imprecision. Discuss both direction and magnitude of any potential bias	9,10	There are limitations to our study … However we cannot rule out cross-reactivity with other unknown alphaviruses.
Interpretation	20	Give a cautious overall interpretation of results considering objectives, limitations, multiplicity of analyses, results from similar studies, and other relevant evidence	9,10	With the serological results, we were able to reconstruct the magnitude of CHIKV transmission that occurred before the 1980s … It will be interesting to see estimates of the FOI and the proportion that remain susceptible after the recent outbreaks in South and Central America.
Generalisability	21	Discuss the generalisability (external validity) of the study results	10	It is not clear why transmission ended … therefore public health agencies should be vigilant for chikungunya cases.
**Other information**				
Funding	22	Give the source of funding and the role of the funders for the present study and, if applicable, for the		Mentioned in submitting process

	original study on which the present article is based

*Give information separately for cases and controls in case-control studies and, if applicable, for exposed and unexposed groups in cohort and cross-sectional studies.

Note: An Explanation and Elaboration article discusses each checklist item and gives methodological background and published examples of transparent reporting. The STROBE checklist is best used in conjunction with this article (freely available on the Web sites of PLoS Medicine at http://www.plosmedicine.org/, Annals of Internal Medicine at http://www.annals.org/, and Epidemiology at http://www.epidem.com/). Information on the STROBE Initiative is available at www.strobe-statement.org.
