# Supplementary material for: Evidence of previous but not current transmission of chikungunya virus in southern and central Vietnam: Results from a systematic review and a seroprevalence study in four locations
Source: PLoS Negl Trop Dis. 2018 Feb 9;12(2):e0006246. doi: 10.1371/journal.pntd.0006246 (PMC5823466; doi:10.1371/journal.pntd.0006246)
Supplement: S1 Text — (DOCX) [file pntd.0006246.s004.docx]

# Supplementary Information: Evidence of previous but not current transmission of chikungunya virus in southern and central Vietnam: Results from a systematic review and a seroprevalence study in four locations

Tran Minh Quan^1*^, Huynh Thi Phuong^1^, Nguyen Ha Thao Vy^1^, Nguyen Thi Le Thanh^1^, Nguyen Thi Nam Lien^2^, Tran Thi Kim Hong^3^, Pham Ngoc Dung^4^, Nguyen Van Vinh Chau^5^, Maciej F Boni^1,6,7^, Hannah E. Clapham^1,6^

1 Oxford University Clinical Research Unit, Wellcome Trust Major Overseas Programme, Ho Chi Minh City, Vietnam

2 Microbiology Department, Hue Central Hospital, Thua Thien Hue province, Vietnam

3 Laboratory Department, Dak Lak General Hospital, Buon Ma Thuot, Vietnam

4 Laboratory Department, An Giang General Hospital, An Giang province, Vietnam

5 Hospital for Tropical Diseases, Ho Chi Minh City, Vietnam

6 Centre for Tropical Medicine and Global Health, Nuffield Department of Medicine, University of Oxford, Oxford, UK

7 Center for Infectious Disease Dynamics, Department of Biology, Pennsylvania State University, USA

* Corresponding author

E-mail: [quantm@oucru.org](mailto:hclapham@oucru.org)

1. **Force of infection estimation:**

The numbers of seropositive and seronegative people are stratified by age groups of 5 years, which are 0-4, 5-9, 10-14, 15-19, 20-24, 25-29, 30-34, 35-39, 40-44, 50-54, 55-59, 60-64, 65-69, and 70+. The likelihood function representing the contribution of a seropositive individual is:

$\boldsymbol{L}_{\boldsymbol{n}}^{\boldsymbol{pos}}\boldsymbol{=1-}\mathbf{exp}\left( \begin{aligned} \boldsymbol{-5}\sum_{\boldsymbol{i=}\boldsymbol{1}}^{\boldsymbol{(}\boldsymbol{n}\boldsymbol{-1)}} \boldsymbol{\lambda}_{\boldsymbol{i}}\boldsymbol{-2.5}\boldsymbol{\lambda}_{\boldsymbol{n}} \end{aligned} \right)$ ( 1 )

Where $L_{n}^{pos}$ is the likelihood function representing the contribution of number of seropositive individual in age group$n$. While $\lambda_{i} (i=1, 2, 3, \ldots14)$ is the average annual FOI in respective year period 2011-2015, 2006-2010, 2001-2005, etc. Also, the last FOI $\lambda_{15}$is defined as the average annual FOI in year period 1931-1945. For example, the likelihood of seropositive individuals in 5-9 age group is$L_{5-9}^{pos}= 1-exp[-(5\lambda_{1}+2.5\lambda_{2})]$. We can intuitively understand this as: the positive individuals in 5-9 age group is affected by the FOI since the time when they were born, which for this age group, is average 7.5 years ago and contain 2 pre-defined year periods: 2011-2015 (which everyone experienced all of) and 2010-2006 (which this group on average experienced half of).

Similarly, the likelihood equation $L_{n}^{neg}$ for a seronegative individual by each age group $n$ is:

$\boldsymbol{L}_{\boldsymbol{n}}^{\boldsymbol{neg}}\boldsymbol{=}\mathbf{exp}\boldsymbol{}\left( \boldsymbol{-5}\sum_{\boldsymbol{i=1}}^{\boldsymbol{(n-1)}} \boldsymbol{\lambda}_{\boldsymbol{i}}\boldsymbol{-2.5}\boldsymbol{\lambda}_{\boldsymbol{n}} \right)$ ( 2 )

We defined the average age for the last age group 70+ to be 77.5 years old. Therefore, the likelihood for the last age group is:

$\boldsymbol{L}_{\boldsymbol{70+}}^{\boldsymbol{pos}}\boldsymbol{=1-}\mathbf{exp}\left( \begin{aligned} \boldsymbol{-5}\sum_{\boldsymbol{i=}\boldsymbol{1}}^{\boldsymbol{14}} \boldsymbol{\lambda}_{\boldsymbol{i}}\boldsymbol{-7.5}\boldsymbol{\lambda}_{\boldsymbol{15}} \end{aligned} \right)$ ( 3 )

And

$\boldsymbol{L}_{\boldsymbol{70+}}^{\boldsymbol{neg}}\boldsymbol{=}\mathbf{exp}\boldsymbol{}\left( \boldsymbol{-5}\sum_{\boldsymbol{i=1}}^{\boldsymbol{14}} \boldsymbol{\lambda}_{\boldsymbol{i}}\boldsymbol{-7.5}\boldsymbol{\lambda}_{\boldsymbol{15}} \right)$ ( 4 )

The total log-likelihood for the whole dataset of seroprevalence in 2015 was calculated as the sum of all log-likelihoods from each seronegative and seropositive in every age group.

To estimate the random variables$\lambda_{i}$, we use RStan package in R [1]. We initiated 4 independent chains for each model, each with the 4000 iterations and 50% burn-in period. The convergence of each chain was inspected visually and numerically (effective sample size and potential scale reduction statistic$\hat{R}$) by bayesplot package in R [2]. We further tried to infer the time period when the last transmission happened by introducing an index$i_{end}$. Every $\lambda_{i}$ before $i_{end}$ are fixed to near 0, hence no transmissions occur:

$\boldsymbol{\lambda}\left[ \boldsymbol{1:}\left( \boldsymbol{i}_{\boldsymbol{end}}\boldsymbol{-1} \right) \right]\boldsymbol{\approx0}$ ( 5 )

The other $\lambda_{i}$ were sampled from a wide uninformative prior distribution:

$\boldsymbol{\lambda}\left[ \boldsymbol{i}_{\boldsymbol{end}}\boldsymbol{:n} \right]\boldsymbol{\sim normal(0, 1000)}$ ( 6 )

All $\lambda_{i}$were constrained to be positive.

Since we have 15 year periods, there are respective 16 values of $i_{end}$ and 16 corresponding models for each location. $i_{end}=1$ means that there is still transmission until today, while $i_{end}=16$ means there are no transmission of CHIKV at all. We compared models within each location by the DIC value. We then find the best model (smallest value) in each location and infer the year periods in which the final transmission is most likely to occur.

1. **Definition of** $\boldsymbol{\lambda}_{\boldsymbol{j,k}}^{\boldsymbol{aff}}$ **and 2 endemic scenarios:**

$\lambda_{j,k}^{aff}$is the FOI that affects each age group $k \left( k=0,1,2, \ldots, 79, 80+ \right)$ in the population in year $j$. Since we do not have any information of endemic situation of CHIKV in Vietnam in 1930 and before, we proposed two scenarios: no transmission before 1930 and endemic transmission before 1930. We defined $\lambda_{j}^{aff}=[\lambda_{j,0}^{aff}, \lambda_{j,1}^{aff}, \ldots,\lambda_{j,80+}^{aff}]$to be the vector of FOI that affects all age groups $k$ in year $j$. In the first scenario we assume the FOI before 1930 was 0 and in the second we assume the FOI before 1930 was the same as in 1931. Hence the FOI of 1930 in the no transmission scenario is$\lambda_{1930}^{aff}=\boldsymbol{0}$ ($\boldsymbol{0}$ is a vector 0 with length equal to number of age groups $k$, which is 81), while we set $\lambda_{1930}^{aff}={[\lambda}_{1931},{2\lambda}_{1931}, ..., {81\lambda}_{1931}]$ in endemic scenario with $\lambda_{1931}$ is the FOI that affect each age group $k$ in the year 1931 and can be estimated from the model. It can be interpreted that the endemic scenario is an extreme case when every people in the population have been affected by the same FOI for their life time. In sequenced years, $\lambda_{\left( j+1 \right)}^{aff}$ will be calculated as:

$\boldsymbol{\lambda}_{\left( \boldsymbol{j+1} \right)}^{\boldsymbol{aff}}\boldsymbol{=}\boldsymbol{\lambda}_{\boldsymbol{j}}^{\boldsymbol{aff,rs}}\boldsymbol{+}\boldsymbol{\lambda}_{\boldsymbol{j+1}}$ ( 7 )

With $\lambda_{j}^{aff,rs}$ is defined as a vector shifted to the right by one element from vector $\lambda_{j}^{aff}$. For example, $\lambda_{1930}^{aff,rs}=[0, \lambda_{1931},{2\lambda}_{1931}, ..., 80\lambda_{1931}]$ is right-shifted from $\lambda_{1930}^{aff}=\left[ \lambda_{1931},{2\lambda}_{1931}, ..., {81\lambda}_{1931} \right]$ in endemic situation. On the other hand, $\lambda_{j+1}$ is the vector with$81$ replicated elements of FOI in year $j+1$ estimated from the model. The reasoning behinds the formula ( 7 ) is that the population cohort of the sequenced year is under the effects of both FOI of the previous year (excepts for the first age group $0$ newly introduced to the population, and the last age group $80+$, which grows out of the population cohort) and the age-independent estimated FOI of the sequenced year.

1. **Posterior distributions of FOI from the best model:**

**
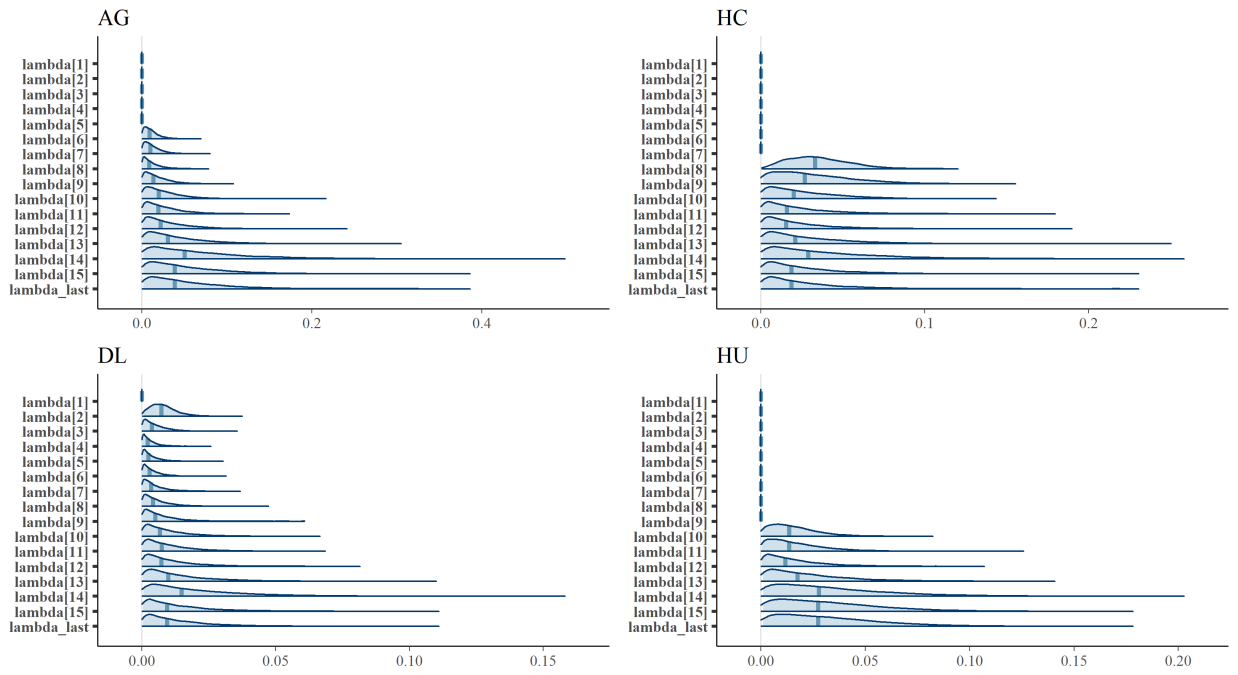
**

**Figure A. The posterior distributions of estimated FOIs from the best model in each place.** There are 15 lambda for 15 predefined time periods (as mentioned in the methodology). The lambda_last was set to be the same as lambda[15] and used to generate the susceptible proportion in endemic scenario. The vertical lines represent the mean of each distribution. Abbreviation: AG: An Giang, HC: Ho Chi Minh city, DL: Dak Lak, HU: Hue. The graph is plotted by bayesplot package [2].

1. **FOIs estimation from model with 1 year block:**

By grouping the people by 5-year age groups, we might lose some information of the exact individuals’ age. In this section, we represent the results for an alternative model with 1 year block, which can capture the finest detail of ages for all individuals. The log likelihood of the positive individual contributes to the 1 years block model is:

$\boldsymbol{L}_{\boldsymbol{n}}^{\boldsymbol{pos}}=\boldsymbol{1}-\mathbf{exp}\left( \begin{aligned} -\sum_{\boldsymbol{i=}\boldsymbol{1}}^{\boldsymbol{n-1}} \boldsymbol{\lambda}_{\boldsymbol{i}}-\boldsymbol{0.5}\boldsymbol{\lambda}_{\boldsymbol{n}} \end{aligned} \right)$ ( 8 )

While $\lambda_{i} (i=1, 2, 3, \ldots70)$ is the FOI in every years from 1946 to 2015. Also, the last FOI $\lambda_{71}$is defined as the average annual FOI in year period 1931-1945.

Similarly, the likelihood equation $L_{n}^{neg}$ for a seronegative individual is:

$\boldsymbol{L}_{\boldsymbol{n}}^{\boldsymbol{neg}}\boldsymbol{=}\mathbf{exp}\boldsymbol{}\left( \boldsymbol{-}\sum_{\boldsymbol{i=1}}^{\boldsymbol{(n-1)}} \boldsymbol{\lambda}_{\boldsymbol{i}}\boldsymbol{-0.5}\boldsymbol{\lambda}_{\boldsymbol{n}} \right)$ ( 9 )

The results of estimated FOI from the model can be shown in Figure B. We also combine the data from both An Giang and Ho Chi Minh since they are all from the southern of Vietnam and appear to have the comparable CHIKV activity. The model with data from all locations is likewise shown. Overall, the results from 1 year block model all share the same features, with the decrement of FOI from the past until recently. This may suggests an endemic activity of the virus in the past, followed by a recent no transmission period in these locations. In addition, the combined results from An Giang and Ho Chi Minh as well as from all locations shrink the credible interval of estimated FOI a little but the trend of FOI over time is identical to the specific location results.


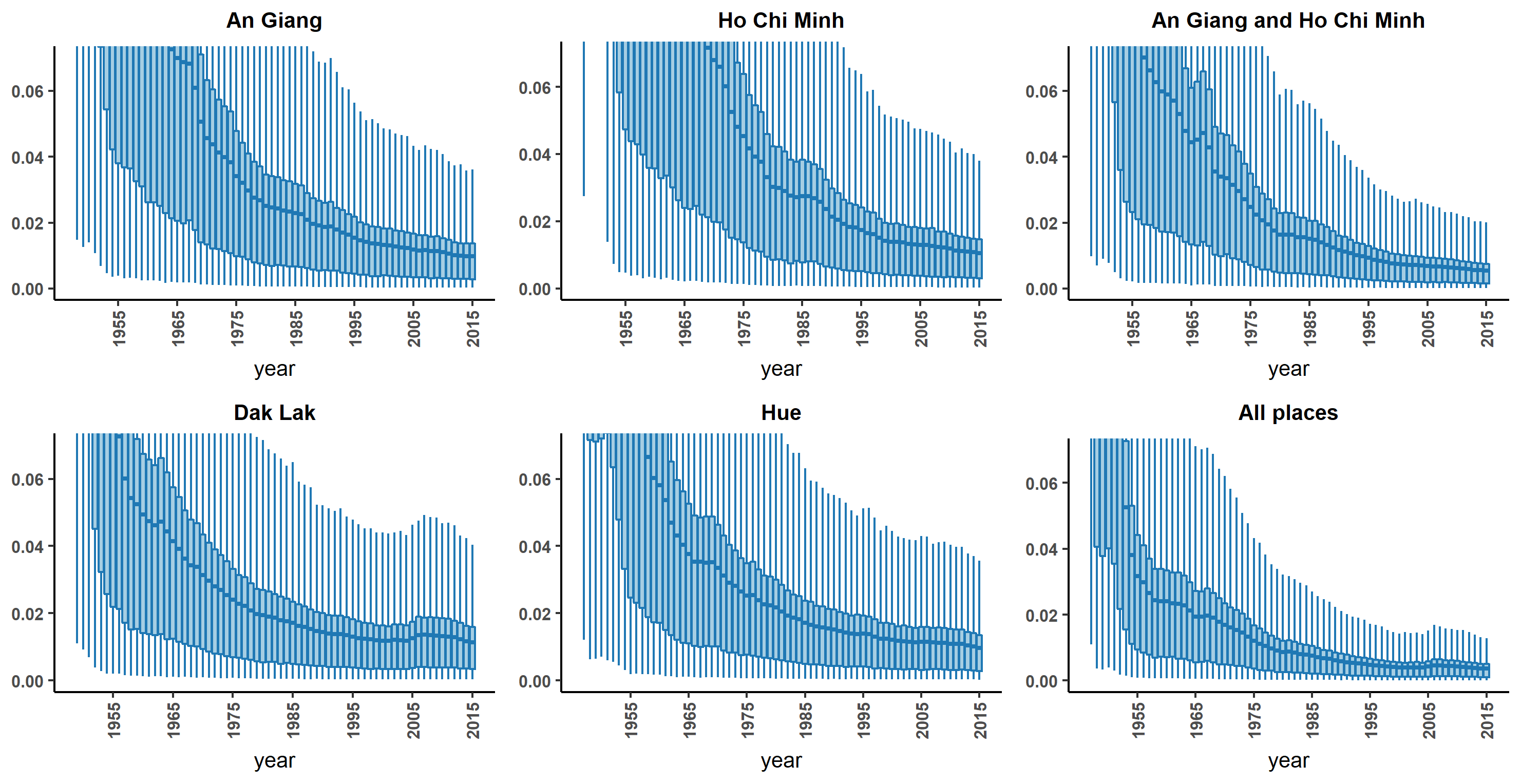


**Figure B. FOI estimates in each year by location from the 1 year block model.** The blue boxplots show the credible intervals with means, 1^st^ quartile and 3^rd^ quartile of annual FOI estimation by time period in each location.

1. **Sensitivity analysis:**

We also compared models from estimated FOI with different blocks of years (1 to 5). In each year block model, the $i_{index}$ was also used to infer the last transmission period. We then selected the best models by DIC values. The log likelihood of the positive individual contributes to the $\boldsymbol{y}$ years block model is:

$L_{n}^{pos}=1-exp\left( \begin{aligned} -\boldsymbol{y}\sum_{\boldsymbol{i=}\boldsymbol{1}}^{\boldsymbol{n-1}} \boldsymbol{\lambda}_{\boldsymbol{i}}-\frac{\boldsymbol{y}}{\boldsymbol{2}}\boldsymbol{\lambda}_{\boldsymbol{n}} \end{aligned} \right)$ ( 10 )

The results of the best models and the models which have DIC values differences < 3 compared to the best one are similar to the previous estimations (Table). In every locations, the model with 1 or 2 years block are not among the best models, suggest that our data may not have enough resolution for FOI estimation in the smaller year block.

**Table. Last transmission year periods inferred from the best models among different year blocks (1 to 5) by each location.**

| **Location** | **Year block** | **Last transmission year period** | **Previous estimations (5 years block)** |
| --- | --- | --- | --- |
| An Giang | 3 | 1983-1986 | 1981-2000 |
|  | 4 | 1980-1999 |  |
|  | 5 | 1981-2000 |  |
| Ho Chi Minh | 4 | 1983-1991 | 1976-1995 |
|  | 5 | 1976-1995 |  |
| Dak Lak | 5 | 2001-2015 | 2001-2015 |
| Hue | 4 | 1968-1971 | 1961-1985 |
|  | 5 | 1961-1985 |  |

**References:**

1. Stan Development Team (2016), *RStan: the R interface to Stan.* R package version 2.14.1.

2. Gabry J (2017), *bayesplot: Plotting for Bayesian models.* R package version 1.2.0.
