# Supplementary material for: Evidence of previous but not current transmission of chikungunya virus in southern and central Vietnam: Results from a systematic review and a seroprevalence study in four locations
Source: PLoS Negl Trop Dis. 2018 Feb 9;12(2):e0006246. doi: 10.1371/journal.pntd.0006246 (PMC5823466; doi:10.1371/journal.pntd.0006246)
Supplement: S2 Text — (DOCX) [file pntd.0006246.s005.docx]

**COMPARISONS OF FOI ESTIMATION, SUSCEPTIBLE PROPORTION OF BEST MODELS**

**
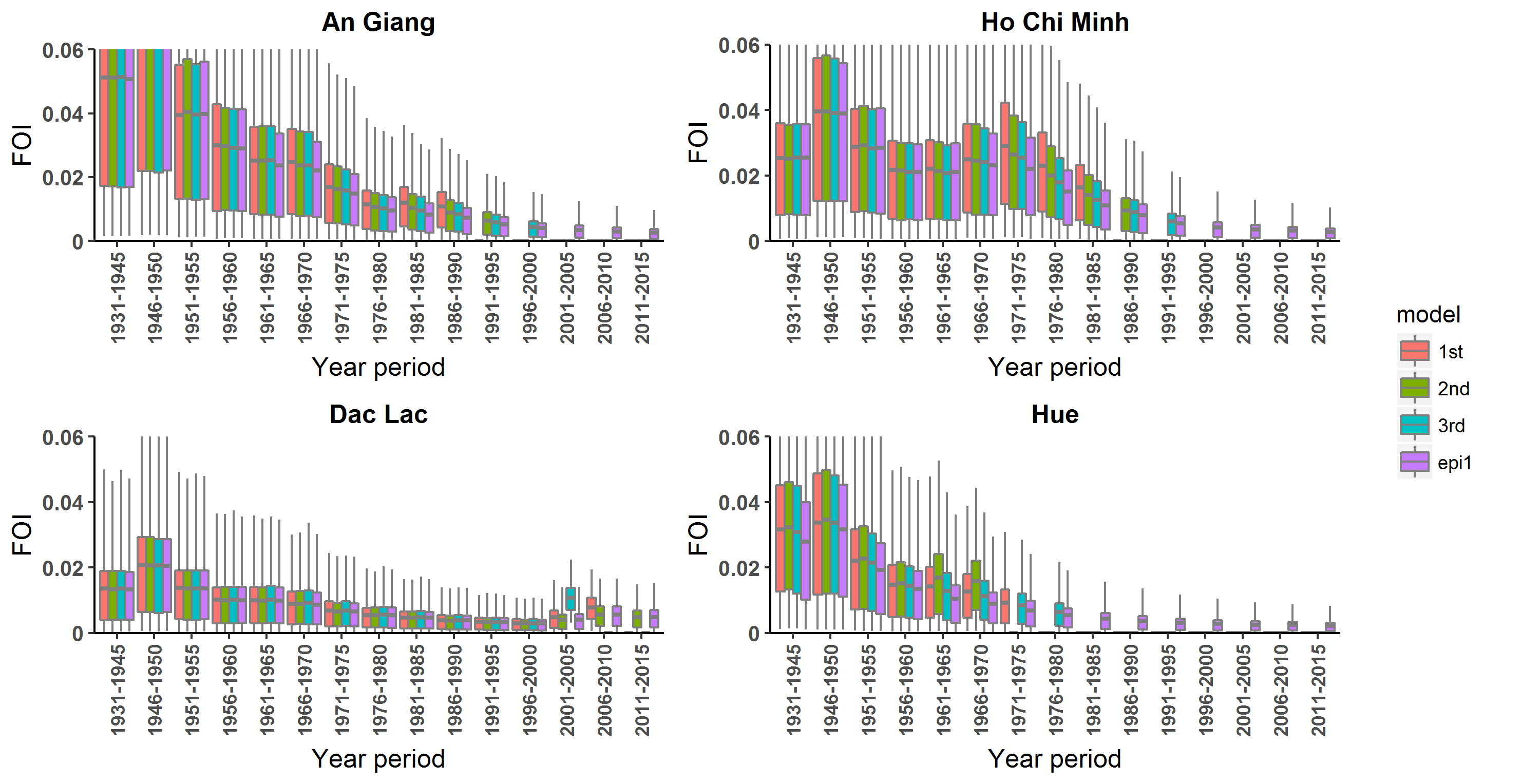
**

**Figure A. FOI estimates in each period by location from the 3 best fit models and the model with** $\boldsymbol{i}_{\boldsymbol{index}}\boldsymbol{=1}$**.** The blue boxplots show the credible intervals with means, 1^st^ quartile and 3^rd^ quartile of annual FOI estimation by time period in each location. The colors of the boxplots represent each model are shown in the legend, with epi1 is the model with $i_{index}=1$.

**
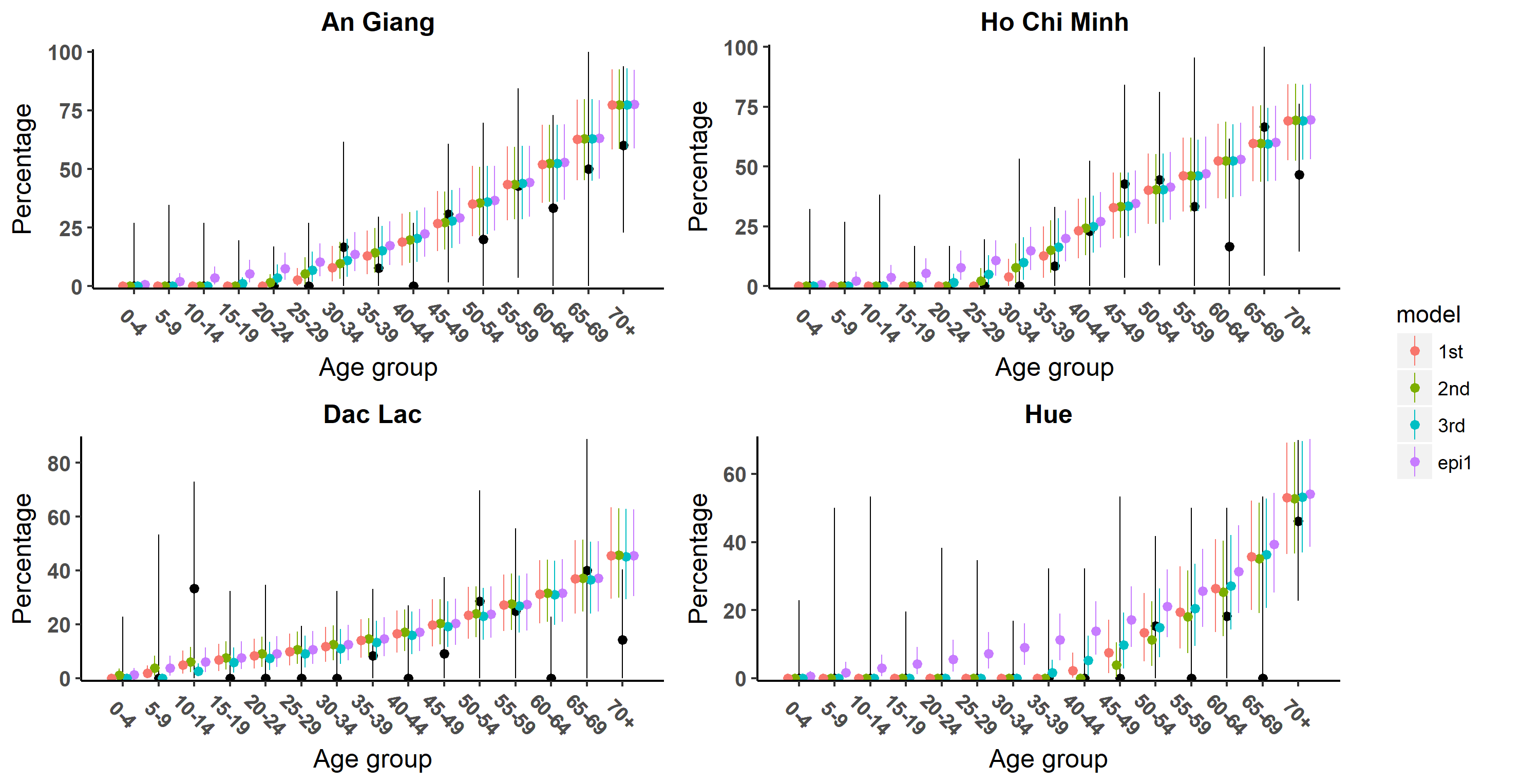
**

**Figure B. Model fit of the 3 best fitting model and the model with** $\boldsymbol{i}_{\boldsymbol{index}}\boldsymbol{=1}$ **to the age-specific seropositive proportion in each location.** The black dots are the seropositive proportion in each age group along with corresponding binomial 95% confidence intervals, corrected by known 90% sensitivity and 90% specificity of the diagnosis test [[1](#_ENREF_1)]. The colored dots are the model output generated from the model run with the mean parameters, along with their 95% credible intervals. The colors of the dots represent each model are shown in the legend, with epi1 is the model with $i_{index}=1$

**
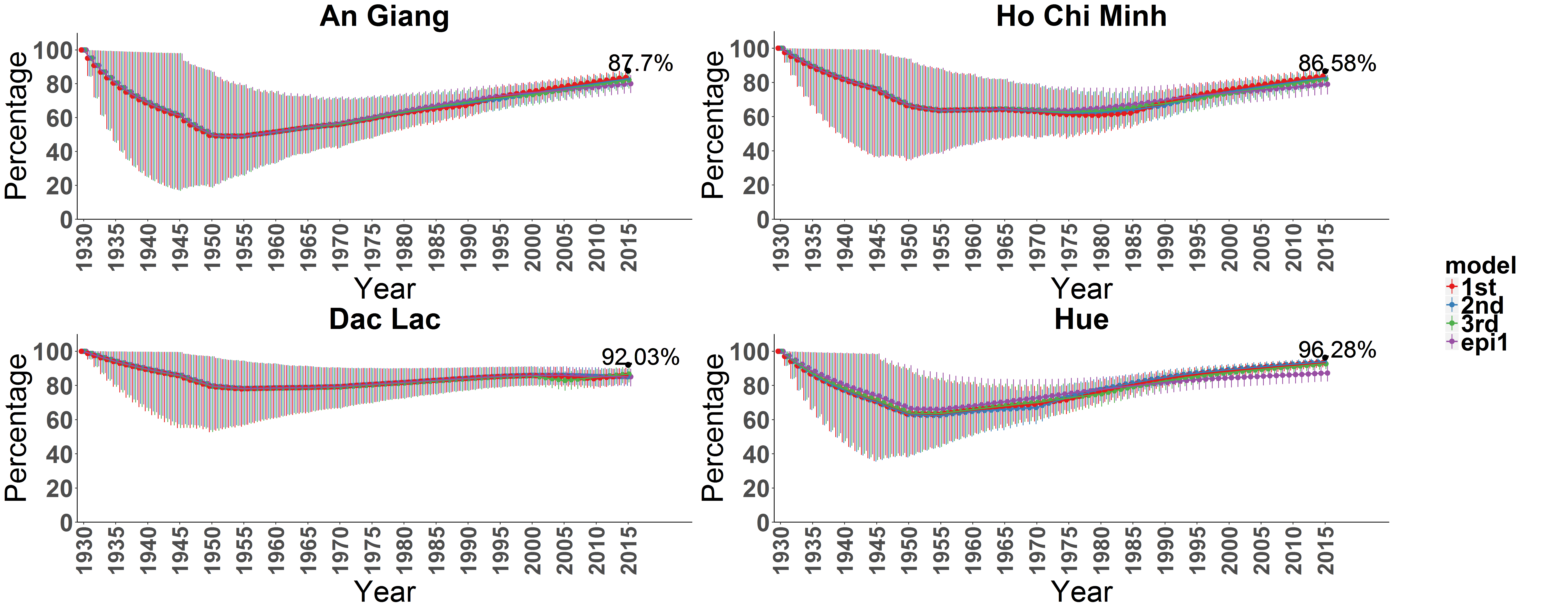
**

**Figure C. The predicted susceptible proportions over time with no endemic transmission before 1930 scenario from the 3 best model and the model with** $\boldsymbol{i}_{\boldsymbol{index}}\boldsymbol{=1}$ **in each location.** In all locations, the boxplots represent the generated proportion with its 95% credible intervals (included 1^st^ quartile, 3^rd^ quartile, and the medians) with the solid lines showing the mean value of each interval. The colors of the boxplots and the solid lines represent each model are shown in the legend, with epi1 is the model with $i_{index}=1$. The black dots are the age-adjusted seropositive proportions from our serosurvey in 2015.

**
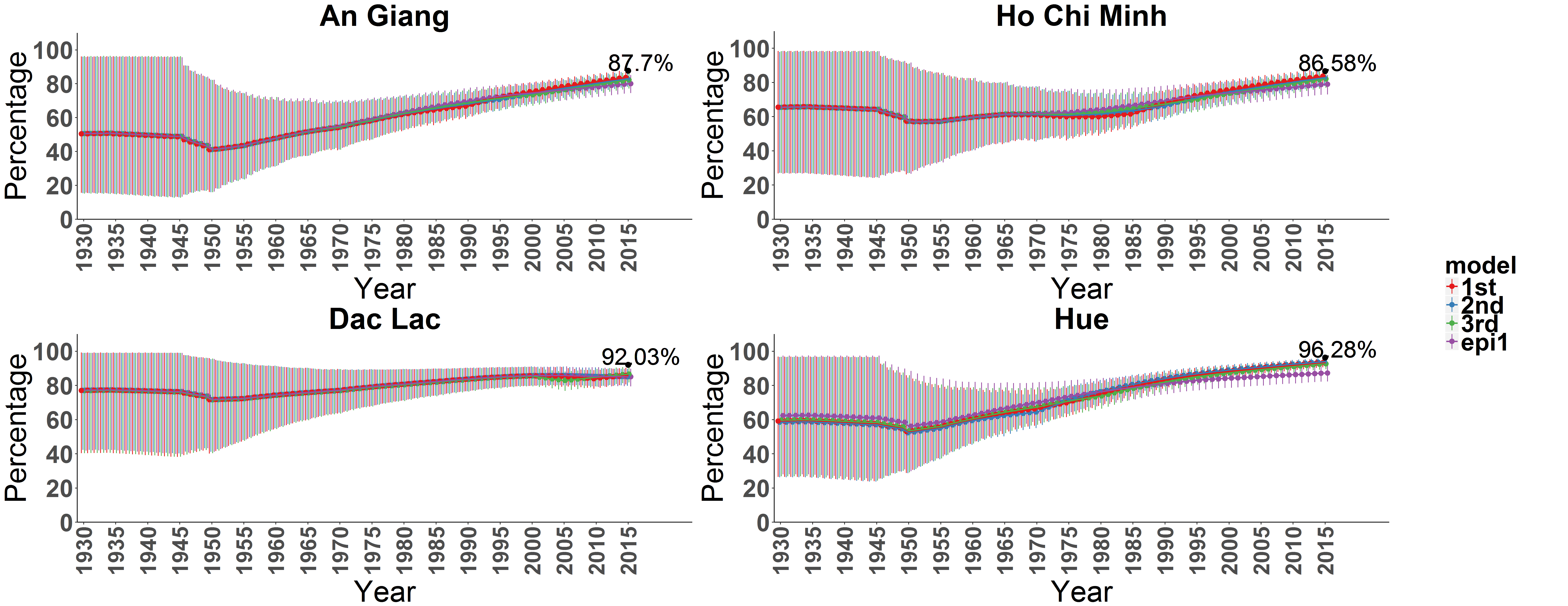
**

**Figure D. The predicted susceptible proportions over time with endemic transmission before 1930 scenario from the 3 best model and the model with** $\boldsymbol{i}_{\boldsymbol{index}}\boldsymbol{=1}$ **in each location.** In all locations, the boxplots represent the generated proportion with its 95% credible intervals (included 1^st^ quartile, 3^rd^ quartile, and the medians) with the solid lines showing the mean value of each interval. The colors of the boxplots and the solid lines represent each model are shown in the legend, with epi1 is the model with $i_{index}=1$. The black dots are the age-adjusted seropositive proportions from our serosurvey in 2015.

The results of second and third best models only slightly different from the best model. Noticeably, the result of susceptible proportion estimation of all models in Dak Lak fail to capture the seropositive at 2015.

The results of the models when $i_{index}=1$ (means there are possibilities that the transmission is still active until 2015) all failed to capture the seropositive proportion in year 2015.

**REFERENCE**

1. Reiczigel J, Foldi J, Ozsvari L. Exact confidence limits for prevalence of a disease with an imperfect diagnostic test. Epidemiol Infect. 2010;138(11):1674-8.
