# Supplementary material for: Evidence of previous but not current transmission of chikungunya virus in southern and central Vietnam: Results from a systematic review and a seroprevalence study in four locations
Source: PLoS Negl Trop Dis. 2018 Feb 9;12(2):e0006246. doi: 10.1371/journal.pntd.0006246 (PMC5823466; doi:10.1371/journal.pntd.0006246)
Supplement: S1 Table — (DOCX) [file pntd.0006246.s006.docx]

**S1 Table. Description of papers included in systematic results.**

| ID | Tittle | Sampled date | Population | Methodology | Results | Notes/Ref |
| --- | --- | --- | --- | --- | --- | --- |
| **PUBMED SEARCH** | | | | | | |
| 1 | Dengue hemorrhagic fever in South Vietnam: report of the 1963 outbreak. | 1) An Giang: August 1963  2) HCM: November 1963 | 1) An Giang: 9 + 2 (fatal) acute hemorrhagic children (2y-13y)  2) Saigon: 8 convalescent stage (3y -11y), no hemorrhagic + 75 normal children (3m -12y). | 1)Virus isolation  2)Serology: HI, CF | An Giang: HI: 5/11; CF: 3/9  Saigon: HI:3/8; CF: 3/8  Normal Saigon: HI: 22/75; CF: 16/75 | [[1](#_ENREF_1)] |
| 2 | [Study of anti-Chikungunya antibodies in Vietnamese children in Saigon]. | 1963 | 472 healthy children of Saigon | Antibody (no other information included) | 148/472 (31.3%) had antibody above or equal 1/40  304/472 (64.4%) had antibody above or equal 1/10 | There is a table of percentage of titer for each group age in the paper.[[2](#_ENREF_2)] |
| 3 | [Hemorrhagic fever in Vietnam in 1964-1965. Serologic study with a brief clinica and epidemiologic note]. | 1964-1965 | 156 hemorrhagic fever patients | Pairs of serum samples (no other information included, maybe HI) | 16 cases with increase in antibody | [[3](#_ENREF_3)] |
| 4 | An analysis of fevers of unknown origin in American soldiers in Vietnam. | April 1^st^ 1966 to August 1^st^ 1966 | 110 U.B soldiers from 93^rd^ Evacuation Hospital, Long Binh, South Vietnam, with symptoms: fever, chill, headache | 1)Virus isolation  2)HI paired sera: 1 for acute, 1 for convalescent | HI rises and Virus isolation (+): 10/110 (9%), no HI positive with Dengue | [[4](#_ENREF_4)] |
| 5 | The distribution and prevalence of group A arbovirus neutralizing antibodies among human populations in Southeast Asia and the Pacific islands. | 1972 | General population in South of Vietnam were submitted for serologic confirmation of suspected cases of dengue | PRNT  Sensitivity: 85-97%; Specificity: 90-98% | 31/130 (24%) | Test for 5 group A arbovirus (CHIKV, RRV, Getah, Bebaru, and Sindbis) in 44 locations in SEA and Pacific island.[[5](#_ENREF_5)] |
| 6 | Retrospective seroepidemiological study of chikungunya infection in South Asia, Southeast Asia and the Pacific region. | 2006 | 44 febrile patients | CHIKV IgM capture ELISA (not commercial test)  CHIKV IgG indirect ELISA  FNRT (focus reduction neutralization test) | ELISA:  IgM: 0%  IgG: 22/44 (50%)  FNRT:  IgM: 0%  IgG: 22/44 (50%)  => CHIKV neutralization confirmed cases: 25% | 748 sera from 6 countries in SEA + 52 from Fiji. [[6](#_ENREF_6)] |
| 7 | Dengue and other common causes of acute febrile illness in Asia: an active surveillance study in children. | Jun 2010- Jul 2011 (292 days) | 32 febrile fever from Tien Giang General Hospital, My Tho City, Tien Giang province | NovaLisa™ Chikungunya IgM μ-capture ELISA: paired sera  Sensitivity: 95.5%, Specificity: 100%. | 19/32 cases IgM positive.  Incidence density 18.5 (11.6-28.6) | Cohort study.  5 countries in SEA. [[7](#_ENREF_7)] |
| 8 | Surveillance of dengue and chikungunya infection in Dong Thap, Vietnam: A 13-month study. | Jan 2012 – Feb 2013 | 131 acute fever, with symptoms compatibles with dengue or chikungunya in Dong Thap general hospital | Reverse transcription multiplex PCR | 0% | Cohort study. [[8](#_ENREF_8)] |
| **VIETNAM JOURNAL OF PREVENTIVE MEDICINE SEARCH** | | | | | | |
| 9 | Dengue and other common causes of acute febrile illness of 2-14 years-old children cohort in My Tho 2011 | 9/2010 to 6/2011 | 150 cohort children from 2-14 at My Tho (Tien Giang) to investigate febrile cases | NovaLisa™ Chikungunya IgM μ-capture ELISA: paired sera | -35 febrile episodes (32 children have at least one febrile fever).  -4 CHIKV (+) cases/ 6 Dengue (+) (66.7%)  -15 CHIKV (+) cases/26 Dengue (-) (57.7%)  -The rate of sero converse between the acute and convalescent in CHIK is 9.3% | Cohort study (a part of the 7^th^ study in Pubmed)[[9](#_ENREF_9)] |
| 10 | Aedes aegypti, Aedes albopictus mosquitoes and risk factors for the diffusion of chikungunya in several provinces in Vietnam, 2012-2014 | 9/2012 to 9/2014 | 1) 558 Human sera: Febrile + one of athragia, myalgia, headache, rash, petechial, hemorrhage + epidemic history of traveling to risk places within 12 days before the onset.  2) 1104 Aedes aegypti, and Aedes albopictus mosquitoes from 5 provinces: Ha Tinh, Hue, Quang Tri, Dac Nong, Long An (which border Laos and Cambodia) | RT-PCR with RNA isolation by QIAamp viral RNA Mini kit.  miScript SYBR Green PCR Kit | 1) Human: 0%  2) Mosquitoes positive: 0.4% in Dac Nong (n=285), 0.2% in Long An (n=580) | [[10](#_ENREF_10)] |
| 11 | Chikungunya virus and it'B vector at 5 cross-border provinces between Viet Nam, Laos and Cambodia, 2012- 2014 | Oct 2012 – September 2014 | The same study as 11^th^ | The same study as 11^th^ | 1)Human: 0%  2)2 mosquitoes from Dak Nong and Long An are positive. These 2 mosquitoes later sequenced but the replicate of RNA is too low to analyse (unpublished) | The same study as 10^th^ [[11](#_ENREF_11)] |
| **NATIONAL LIBRARY OF VIETNAM – PHD THESIS STORAGE** | | | | | | |
| 12 | Situation of Hemorrhagic Dengue fever in Southern Vietnam from 1975 to 1990, epidemic data, virology and preventive methods | 1)1978-1982  2)1979 | 1) Mosquitoes, Human blood from Southern Vietnam  2) Healthy human from 4 locations: HCM city (Binh Thanh, D1), Vung Tau, My Tho, Minh Hai | Virus isolation  HI | 1) 12 cases with positive virus isolation from 1978-1982: 6 hemorrhagic fever children, 1 Ae.Aegypti, 1 Ae.Albopictus, 4 C.fatigans  2)Antigen positive in 4 locations | [[12](#_ENREF_12)] |
| 13 | Investigating the circulation of human induced disease viruses from bat in Vietnam | 2006-2009 | 549 bats from 6 species at Hoa Binh, Tuyen Quang, Bac Giang, Quang Binh, Dak Lak, Dak Nong. | ELISA IgG  Neutralizing test (NT50) | ELISA IgG for CHIKV: 11 samples (2.0%): 2 from Charephone plicata at Bắc Giang; 9 from Rousettus leschenaulti at Hòa Bình. However, all of these samples are negative to NT50 => cross activity? | [[13](#_ENREF_13)] |
| **ADDITIONAL PAPERS** | | | | | | |
| 14 | Applying the RT-PCR assay to identify chikungunya virus in hemorrhagic fever patients | 2009 | 50 patients who suffered from acute hemorrhagic fever from 2009 at Thanh Tri, Ha Noi. The admission symptoms include high fever, rash skin, hemorrhage. | A specific and sensitive multi-RT-PCR assay was used as a tool to detect CHIKV/DENV | 4/50 cases were positive with CHIKV , later be confirmed by gene sequencing  3/50 cases were co-infection with CHIKV/DENV | [[14](#_ENREF_14)] |
| 15 | Chikungunya and Zika Virus Cases Detected Against a Backdrop of Endemic Dengue Transmission in Vietnam | 2012 | 8105 children (1-15 years old) with fever of less than 72 hours and dengue-like symptoms | RT-PCR | 3 (+) cases in Bind Duong province.  1 (+) case in Ho Chi Minh City.  All of the strains are closely related to Cambodia strain in 2011. | [[15](#_ENREF_15)] |
| **PRO-MED SEARCH** | | | | | | |
| 16 | VIET NAM (HANOI) SUSPECTED | 22^nd^ Oct 2009 | People in Hanoi |  | 60% of patients with classis dengue symptoms have (-) test for dengue. | [[16](#_ENREF_16)] |
| **GOOGLE SEARCH** | | | | | | |
| 17 | Secondary Dengue infection is more severe | 2010 | People in middle regions, Tay Nguyen. |  | National Institute of hygiene and Epidemiology defined 15 patients with Dengue like symptom but negative to Dengue and positive to CHIKV | [[17](#_ENREF_17)] |
| 18 | The search of Zika virus in Vietnam | Early 2016 | 83 Zika-alike symptoms people from southern Vietnam |  | 9 positive cases of CHIKV in Can Tho | [[18](#_ENREF_18)] |
| 19 | The finding of 56 mosquitoes positive with Zika in VN | 4/2016 | Mosquitoes in Nha Trang province |  | National Institute of Hygiene and Epidemiology: 0% positive with CHIKV | [[19](#_ENREF_19)] |

**REFERENCE**

1. Halstead SB, Voulgaropoulos EM, Tien NH, Udomsakdi S. Dengue hemorrhagic fever in South Vietnam: report of the 1963 outbreak. The American journal of tropical medicine and hygiene. 1965;14(5):819-30.

2. Vu Qui D, Nguyen-Thi K-T, Ly Quoc B. [Study of anti-Chikungunya antibodies in Vietnamese children in Saigon]. Bull Soc Pathol Exot Filiales. 1967;60(14):353-9.

3. Vu Qui D, Nguyen-Thi K-T. [Hemorrhagic fever in Vietnam in 1964-1965. Serologic study with a brief clinica and epidemiologic note]. Bull Soc Pathol Exot Filiales. 1967;60(1):21-33.

4. Deller JJ, Jr., Russell PK. An analysis of fevers of unknown origin in American soldiers in Vietnam. Ann Intern Med. 1967;66(6):1129-43.

5. Tesh RB, Gajdusek DC, Garruto RM, Cross JH, Rosen L. The distribution and prevalence of group A arbovirus neutralizing antibodies among human populations in Southeast Asia and the Pacific islands. The American journal of tropical medicine and hygiene. 1975;24(4):664-75.

6. Ngwe Tun MM, Inoue S, Thant KZ, Talemaitoga N, Aryati A, Dimaano EM, et al. Retrospective seroepidemiological study of chikungunya infection in South Asia, Southeast Asia and the Pacific region. Epidemiol Infect. 2016;144(11):2268-75.

7. Capeding MR, Chua MN, Hadinegoro SR, Hussain, II, Nallusamy R, Pitisuttithum P, et al. Dengue and other common causes of acute febrile illness in Asia: an active surveillance study in children. PLoS Negl Trop Dis. 2013;7(7):e2331.

8. Kim Lien PT, Briant L, Tang TB, Trang BM, Gavotte L, Cornillot E, et al. Surveillance of dengue and chikungunya infection in Dong Thap, Vietnam: A 13-month study. Asian Pacific Journal of Tropical Medicine. 2016;9(1):39-43.

9. Quang L. Ch. HVTQ, Day Tr. C., Mai Ng. Th. Nh., Hai D. Th., Huu Tr. Ng. [Dengue and other common causes of acute febrile illness of 2-14 years-old children cohort in My Tho 2011]. Vietnam journal of preventive medicine. 2013;XXIII(10):6.

10. Lien Th. K. Pham DTV, Phong V. Tran, Yen Th. Nguyen, Dong D. Tran, Soai V. Nguyen, Cuong C. Tran, Laurent Gavotte, Laurence Briant, Roger Frutos, Duong Nh. Tran. [Aedes Aegypti, Aedes Albopictus mosquitoes and risk factors for the diffusion of Chikungunya in several provinces in Vietnam, 2012-2014]. Vietnam Journal of Preventive Medicine. 2016;XXVI(10):10.

11. Phong V. Tran DTV, Duc H. M., Le Ng. H., Yen Th. Nguyen, Dong D. Tran, Cuong C. Tran, Anh D. Th. V., Tu Tr. C., Soai V. Nguyen, Son Tr. H., Anh Ng. Th. M., Minh H. D., Hang Ng. M., Duong Tr. Nh. [Chikungunya Virus And It's Vector At 5 Cross-Border Provinces Between Vietnam, Laos And Cambodia, 2012 - 2014.]. Vietnam journal of preventive medicine. 2016;XXV(12 + 13):9.

12. Do HQ. [The situation of Dengue epidiemic in the Southern of Vietnam from 1975 to 1990, epidemiology, virology, and preventive methods] [PhD Thesis]. Ho Chi Minh City: University of Medicine and Pharmacy in Ho Chi Minh City; 1991.

13. Nguyen TTT. [A study of the circulation of some disease-induced viruses on human in Vietnam]. Ha Noi - Vietnam: Hanoi University of Natural Science; 2013.

14. Nghia V. X. UND, Thuy Ng. Th., Vien Ng. Tr. Applying the rt-pcr assay to identify chikungunya virus in hemorrhagic fever patients2010.

15. Quyen NTH, Kien DTH, Rabaa M, Tuan NM, Vi TT, Tan LV, et al. Chikungunya and Zika Virus Cases Detected Against a Backdrop of Endemic Dengue Transmission in Vietnam. The American journal of tropical medicine and hygiene. 2017:-.

16. ProMED-mail. Chikungunya virus, humans - Viet Nam (Ha Noi). ProMED-mail 2009.22 October: 20091022.3633.

17. Hien N. [Secondary Dengue infection is more severe] 2010. Available from: <http://vtc.vn/mac-sxh-lan-thu-2-nang-va-de-tu-vong-hon-lan-dau-d20234.html>.

18. Phuong N. [Viet Nam is looking for Zika virus]: Vnexpress; 2016. Available from: <http://suckhoe.vnexpress.net/tin-tuc/suc-khoe/viet-nam-truy-tim-virus-zika-3361736.html>.

19. Phuong C. [The finding of 56 mosquitoes positive to Zika virus in Nha Trang]: Baomoi; 2016. Available from: <http://www.baomoi.com/nha-trang-56-ca-the-muoi-van-nhiem-virus-zika/c/20584519.epi>.
