# Supplementary material for: Evidence of previous but not current transmission of chikungunya virus in southern and central Vietnam: Results from a systematic review and a seroprevalence study in four locations
Source: PLoS Negl Trop Dis. 2018 Feb 9;12(2):e0006246. doi: 10.1371/journal.pntd.0006246 (PMC5823466; doi:10.1371/journal.pntd.0006246)
Supplement: S2 Table — (DOCX) [file pntd.0006246.s007.docx]

The summarized results of negative, borderline, and positive cases are in S2 Table. The table also contain information of the age demographic for each location. The ANOVA test were performed for the age data of 4 places and drawn the result of p-value = 0.063, which means there is no statistical difference. Furthermore, we did the Fisher’s exact test on the results of patients (include negative, borderline, positive test results) stratified by wards at admission in each site, which gives the p-value of 0.8447, 0.06645, 0.07458 for Ho Chi Minh, Dac Lac, and Hue respectively. Hence, there are also no biases of patients’ ward admission in our data. An Giang was not included in our analysis since it only has one ward at admission.

**S2 Table. The summarized results table of negative (N), borderline (B), positive (P), and total (T) cases by total data, sex, ward at admission, also the age demographic (Minimum, maximum, mean, median, and standard deviation values) for each location.**

| **Location** | **An Giang** | | | | **Ho Chi Minh** | | | | **Dac Lac** | | | | **Hue** | | | |
| --- | --- | --- | --- | --- | --- | --- | --- | --- | --- | --- | --- | --- | --- | --- | --- | --- |
| **Total data** | | | | | | | | | | | | | | | | |
| **Test result** | **N** | **B** | **P** | **T** | **N** | **B** | **P** | **T** | **N** | **B** | **P** | **T** | **N** | **B** | **P** | **T** |
|  | 112 | 4 | 21 | 137 | 108 | 6 | 22 | 136 | 123 | 0 | 14 | 137 | 118 | 2 | 16 | 136 |
| **Sex** | | | | | | | | | | | | | | | | |
| **Test result** | **N** | **B** | **P** | **T** | **N** | **B** | **P** | **T** | **N** | **B** | **P** | **T** | **N** | **B** | **P** | **T** |
| **Female** | 71 | 3 | 12 | 86 | 67 | 4 | 12 | 83 | 53 | 0 | 6 | 59 | 71 | 2 | 8 | 81 |
| **Male** | 41 | 1 | 9 | 51 | 41 | 2 | 10 | 53 | 70 | 0 | 8 | 78 | 47 | 0 | 8 | 55 |
| **Ward at admission** | | | | | | | | | | | | | | | | |
| **Test result** | **N** | **B** | **P** | **T** | **N** | **B** | **P** | **T** | **N** | **B** | **P** | **T** | **N** | **B** | **P** | **T** |
| **Out patient** | 112 | 4 | 21 | 137 | 0 | 0 | 0 | 0 | 74 | 0 | 12 | 86 | 79 | 2 | 10 | 91 |
| **General Infectious Disease** | 0 | 0 | 0 | 0 | 55 | 5 | 17 | 77 | 0 | 0 | 0 | 0 | 7 | 0 | 0 | 7 |
| **Hepatitis** | 0 | 0 | 0 | 0 | 1 | 0 | 0 | 1 | 2 | 0 | 0 | 2 | 0 | 0 | 0 | 0 |
| **Respiratory/chest** | 0 | 0 | 0 | 0 | 2 | 1 | 0 | 3 | 0 | 0 | 0 | 0 | 1 | 0 | 2 | 3 |
| **Gastroenterology** | 0 | 0 | 0 | 0 | 3 | 0 | 0 | 3 | 0 | 0 | 0 | 0 | 5 | 0 | 1 | 6 |
| **General Surgical ward** | 0 | 0 | 0 | 0 | 0 | 0 | 0 | 0 | 1 | 0 | 0 | 1 | 1 | 0 | 1 | 2 |
| **Trauma/Orthopedics** | 0 | 0 | 0 | 0 | 0 | 0 | 0 | 0 | 9 | 0 | 0 | 9 | 0 | 0 | 0 | 0 |
| **Intensive care unit** | 0 | 0 | 0 | 0 | 0 | 0 | 0 | 0 | 18 | 0 | 1 | 19 | 1 | 0 | 1 | 2 |
| **Other** | 0 | 0 | 0 | 0 | 47 | 0 | 5 | 52 | 15 | 0 | 1 | 16 | 24 | 0 | 1 | 25 |
| **Unknown** | 0 | 0 | 0 | 0 | 0 | 0 | 0 | 0 | 4 | 0 | 0 | 4 | 0 | 0 | 0 | 0 |
| **Age demographic** | | | | | | | | | | | | | | | | |
| **\|Min, Max\|, \Median, Mean\, Std** | \|1.5, 85.5\|, \35.5, 36.3\, 21.54 | | | | \|1.6, 91.7\|, \36.7, 37.1\, 22.92 | | | | \|1.3, 89.5\|, \39.5, 40.2\, 23.08 | | | | \|1.1, 90.5\|, \43, 43.1\, 24.12 | | | |
