# Supplementary material for: Evidence of previous but not current transmission of chikungunya virus in southern and central Vietnam: Results from a systematic review and a seroprevalence study in four locations
Source: PLoS Negl Trop Dis. 2018 Feb 9;12(2):e0006246. doi: 10.1371/journal.pntd.0006246 (PMC5823466; doi:10.1371/journal.pntd.0006246)
Supplement: S3 Table — (DOCX) [file pntd.0006246.s008.docx]

**S3 Table. Demographic of 3 positive children from Dak Lak province.**

| **AGE** | **GENDER** | **DATE COLLECTED** | **WARD** | **NovaTec_Units** |
| --- | --- | --- | --- | --- |
| 10 | Female | October 27^th^ 2015 | Outpatient | 34.31579 |
| 10 | Male | August 26^th^ 2015 | Outpatient | 36.04167 |
| 13 | Female | August 26^th^ 2015 | Outpatient | 28.23684 |
